# Leukemia-expanded splenic CD81^+^ erythroblasts potentiate disease progression in mice by reshaping leukemic cell metabolism

**DOI:** 10.1172/JCI193082

**Published:** 2025-12-15

**Authors:** Yue Li, Jiaxuan Cao, Jingyuan Tong, Peixia Tang, Haoran Chen, Guohuan Sun, Zining Yang, Xiaoru Zhang, Fang Dong, Shangda Yang, Jie Gao, Xiangnan Zhao, Jinfa Ma, Di Wang, Lei Zhang, Lin Wang, Tao Cheng, Hui Cheng, Lihong Shi

**Affiliations:** 1 State Key Laboratory of Experimental Hematology, National Clinical Research Center for Blood Diseases, Haihe Laboratory of Cell Ecosystem, Tianjin Institutes of Health Science, Institute of Hematology and Blood Diseases Hospital, Chinese Academy of Medical Sciences and Peking Union Medical College, Tianjin, China.; 2 Department of Hematology, Beijing Anzhen Hospital, Capital Medical University, Beijing, China.; 3 Department of Oncology, Tianjin Union Medical Center, The First Affiliated Hospital of Nankai University, Tianjin, China.; 4 State Key Laboratory of Common Mechanism Research for Major Disease, Department of Pharmacology, Institute of Basic Medical Sciences, Chinese Academy of Medical Sciences and Peking Union Medical College, Beijing, China.

**Keywords:** Cell biology, Hematology, Metabolism, Leukemias

## Abstract

During the progression of acute myeloid leukemia (AML), extramedullary hematopoiesis (EMH) compensates for impaired bone marrow hematopoiesis. However, the specific cellular dynamics of EMH and its influence on AML progression remain poorly understood. In this study, we identified a substantial expansion of the CD81^+^ erythroblast subpopulation (CD81^+^ Erys) in the spleens of AML mice, which promoted AML cell proliferation and reduced survival. Mechanistically, CD81^+^ Erys secrete elevated levels of macrophage migration-inhibitory factor (MIF), which interacted with the CD74 receptor on AML cells, activating the mTORC1 signaling pathway and upregulating *Egln3*. Consequently, AML cells cocultured with CD81^+^ Erys exhibited reprogrammed phospholipid metabolism, characterized by an increased phospholipid-to-lysophospholipid ratio. Modulating this metabolic shift, either by supplementing exogenous lysophospholipids or depleting *Egln3* in AML cells, restored the phospholipid balance and mitigated the protumorigenic effects induced by CD81^+^ Erys. Overall, our findings elucidate the molecular crosstalk between erythroblasts and AML cells, extend our insights into the mechanisms driving AML progression, and suggest potential therapeutic strategies.

## Abstract 2

CD81^+^ erythroblasts expand during AML and interact with malignant cells to activate the MIF/CD74-mTORC1-*Egln3* axis, ultimately driving disease progression by reshaping leukemic cell metabolism.

## Graphical abstract

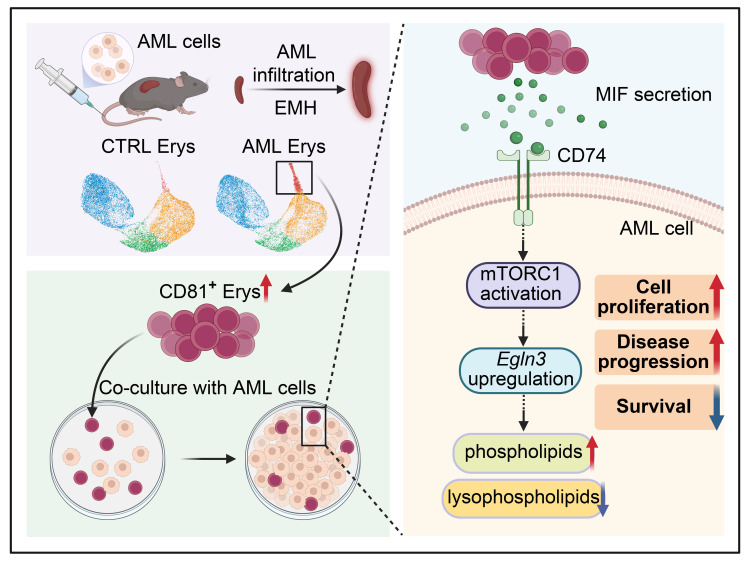

## Introduction

Acute myeloid leukemia (AML) is a malignancy of hematopoietic progenitor cells that profoundly alters its microenvironment, affecting both hematopoietic and nonhematopoietic cell populations. In murine leukemia models, changes in the BM microenvironment include an increase in microvascular density with functional impairment, altered cytokine secretion by mesenchymal stem cells (MSCs), and depletion of osteoblasts (1–5). These changes, combined with stress from AML blasts, force BM hematopoietic stem cells into a more quiescent state. This shift markedly reduces the numbers of hematopoietic stem and progenitor cells and their progeny in the BM, leading to global hematopoietic failure and persistent dysfunction (6, 7).

To compensate for impaired BM hematopoiesis during AML progression, extramedullary hematopoiesis (EMH) is activated in the spleen. It has been reported that splenocytes promote the migration and enhance the pathogenicity of leukemic cells within this specialized microenvironment (8). Clinically, splenomegaly is significantly associated with lower rates of complete remission in patients with AML (9). A higher splenic volume at the time of hematopoietic stem cell transplantation has been identified as an independent predictor of adverse outcomes in patients with AML, including reduced overall survival and increased non-relapse mortality (10). Beyond AML, the cellular components of the spleen, comprising both hematopoietic and nonhematopoietic cells, have been shown to facilitate the progression of various solid tumors (11–14). Although the involvement of splenic EMH in tumor progression has been documented, its specific impact on the progression of hematological malignancies remains largely unexplored. Therefore, a comprehensive analysis of splenic EMH and changes in cellular composition during tumor progression could provide insights into the mechanisms of AML pathogenesis and identify potential therapeutic strategies for AML and other hematological malignancies.

In addition to conventional microenvironmental cells such as immune cells and (MSCs), nucleated erythroid cells have garnered increasing attention as critical components of the splenic microenvironment, exhibiting functions beyond their canonical role in oxygen transport. During development, nucleated erythroid cells contribute to fetomaternal tolerance, bile duct development, and the colonization of the neonatal gut microbiome (15–21). In advanced solid tumors, erythroid lineage cells mediate immunosuppressive effects by inhibiting CD8^+^ T cells, undergoing erythroid-to-myeloid conversion (22, 23), or secreting cytokines to promote tumor metastasis (24, 25). In our previous investigation, we unveiled the heterogeneity of erythroblasts and identified a subset with immunomodulatory properties (26). Moreover, unique cell populations can emerge under stress conditions (27). Therefore, as a key component of the spleen, studying alterations in the erythroid compartment during tumor progression may provide valuable insights into the pathogenesis of malignant diseases, including AML.

Herein, we aim to (a) comprehensively dissect the non-AML hematopoietic lineage cellular components within the splenic microenvironment during AML progression; (b) analyze cellular interactions within the AML splenic microenvironment and their impact on AML progression, with a particular focus on nucleated erythroid cells; and (c) determine whether disrupting specific branches within this cellular network can halt AML progression.

## Results

### An erythroblast population is markedly expanded in the spleens of AML mice.

To explore the effects of AML cell infiltration on hematopoiesis, we analyzed alterations in multiple hematopoietic compartments within the BM, and 2 EMH sites — the liver and spleen — using a nonirradiated AML mouse model driven by the human MLL-AF9 fusion protein (28) (Supplemental Figure 1A; supplemental material available online with this article; https://doi.org/10.1172/JCI193082DS1). When AML cells composed 60% of the peripheral blood (PB) cells in leukemic mice, we observed a significant reduction in the number of all non-AML hematopoietic lineage cells, including CD45^−^ cells, B220^+^ B cells, CD4^+^ T cells, CD8^+^ T cells, Gr1/CD11b^+^ myeloid cells, and F4/80^+^ macrophages, in both the BM (1.2 × 10^8^ vs. 1.3 × 10^6^ cells in control [CTRL] vs. AML, respectively) and liver (4.6 × 10^8^ vs. 7.5 × 10^7^ cells in CTRL vs. AML, respectively) (Supplemental Figure 1B). In the BM, the proportions of myeloid cells and CD4^+^ T cells increased, whereas the cell proportions in the liver remained largely unchanged, except for CD4^+^ T cells (Supplemental Figure 1B).

**Figure 1:**
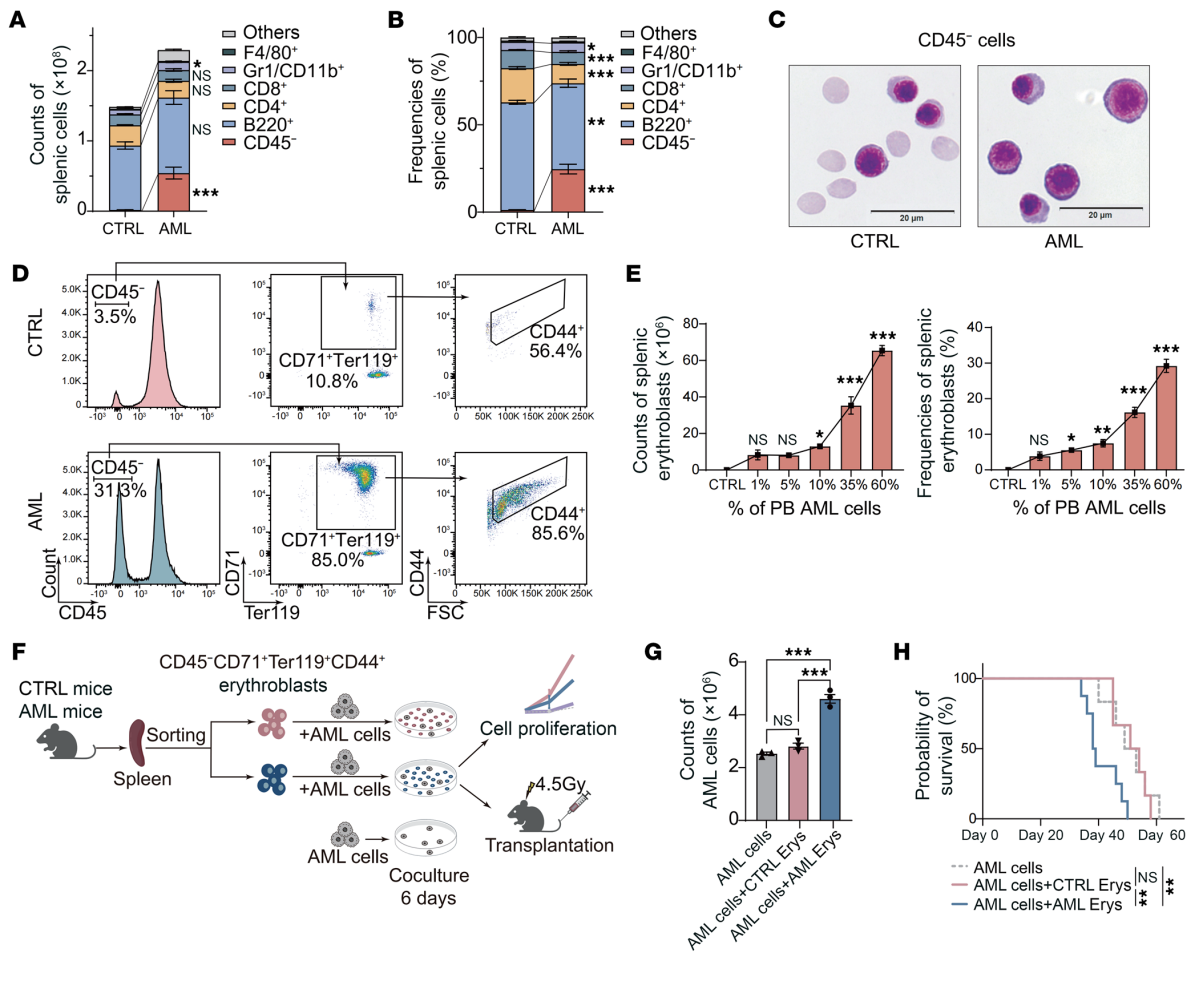
An erythroblast population is markedly expanded in the spleens of AML mice. (**A** and **B**) Cell counts (**A**) and frequencies (**B**) of CD45^−^ cells, B220^+^ B cells, CD4^+^ T cells, CD8^+^ T cells, Gr1/CD11b^+^ myeloid cells, F4/80^+^ macrophages, and other non-AML cell populations in the spleens of control (CTRL) mice and mice with advanced AML (*n* = 4); advanced AML was defined as the presence of 50%–60% AML cells in PB. (**C**) Wright-Giemsa staining of CD45^−^ cells from the spleens of CTRL mice and mice with advanced AML. Scale bar: 20 μm. (**D**) Flow cytometric analysis of CD71 (erythroid precursor marker), Ter119 (erythrocyte marker), and CD44 (differentiation marker) expression in CD45^−^ cells from the spleens of CTRL mice and mice with advanced AML. (**E**) Cell counts and frequencies of CD45^−^CD71^+^Ter119^+^CD44^+^ erythroblasts in the spleens of CTRL mice and AML mice at different disease stages (*n* = 3). (**F**) A schematic illustrating the experimental design: AML cells were cocultured with CD45^−^CD71^+^Ter119^+^CD44^+^ erythroblasts isolated from the spleens of CTRL mice or mice with advanced AML, or cultured alone. After culturing, the AML cells were isolated and injected into irradiated (4.5 Gy) mice. (**G**) Cell counts of AML cells obtained after 6 days of culture alone or coculture (*n* = 3). (**H**) Survival analysis of mice i.v. injected with cultured AML cells (*n* ≥6). Data are presented as the mean ± SEM. The results shown are representative of 1 of 3 independent experiments with consistent trends. *P < 0.05, **P < 0.01, and ***P < 0.001, by 2-tailed, unpaired *t* test (**A** and **B**), 1-way ANOVA (**E** and **G**), and log-rank test (**H**).

In contrast, the number of non-AML hematopoietic cells in the spleen was markedly increased in AML mice (1.5 × 10^8^ vs. 2.2 × 10^8^ cells in CTRL vs. AML, respectively) (Figure 1A), consistent with the observed splenomegaly (Supplemental Figure 1C). Intriguingly, further investigation revealed that the expansion was primarily driven by a marked increase in CD45^−^ cells (Figure 1B). To further characterize the CD45^−^ compartment, we purified CD45^−^ cells via FACS and performed Wright-Giemsa staining. Morphological assessment indicated a strong resemblance to erythroid lineage cells (Figure 1C). Using erythroid lineage–specific markers Ter119, CD71, and CD44 (29), we confirmed that the majority of CD45^−^ cells from AML mice spleen were nucleated erythroid cells, which aligned with the Wright-Giemsa staining results (Figure 1D). Thus, we demonstrated that the significantly expanded CD45^−^ cell population in the spleens of AML mice consisted almost exclusively of erythroblasts (CD45^−^CD71^+^Ter119^+^CD44^+^).

To scrutinize the kinetics of erythropoiesis in the spleen, BM, and PB during AML progression, we monitored key indices associated with erythroid lineage alterations. We found that erythropoiesis in the BM was almost completely impaired, as very few erythroblasts were detected at the advanced disease stage (Supplemental Figure 1D). In contrast, both the cell counts and frequencies of erythroblasts gradually increased in the spleen (Figure 1E), likely originating from expanded erythroid progenitors (30) (Supplemental Figure 1, E and F). Based on this observation, we hypothesized that the severe impairment of erythropoiesis in the BM was partially compensated by a stress erythropoiesis response in the spleen, leading to mild anemia in the PB (Supplemental Figure 1G).

Given the substantial expansion of erythroblasts in the spleens of AML mice, we next sought to investigate their functional role. We cocultured AML cells with erythroblasts from either CTRL mice or mice with advanced-stage AML (i.e., with 50%–60% AML cells in PB) (Figure 1F). It turned out that AML cells cocultured with AML erythroblasts (AML Erys) exhibited enhanced proliferative capacity compared with those cocultured with CTRL erythroblasts (CTRL Erys) or cultured alone (Figure 1G and Supplemental Figure 1H). Notably, mice injected with FACS-purified AML cells that had been cocultured with AML Erys exhibited significantly shortened survival (Figure 1H). These results suggest that the accumulation of AML-expanded erythroblasts in the spleen may contribute to AML progression.

To investigate whether this pro-proliferative effect was confined to erythroblasts derived from the spleens of AML mice, we cocultured AML cells with erythroblasts isolated from the spleens of other mouse models known to induce splenic EMH, such as polycythemia vera (PV), erythropoietin-treated (EPO-treated), and phenylhydrazine-treated (PHZ-treated) mice (Supplemental Figure 1I). Interestingly, although splenic EMH occurred across multiple stress erythropoiesis models, only AML splenic erythroblasts uniquely promoted AML progression (Supplemental Figure 1, J–L).

### CD81^+^ Erys are notably enriched in the spleens of AML mice.

**Figure 2:**
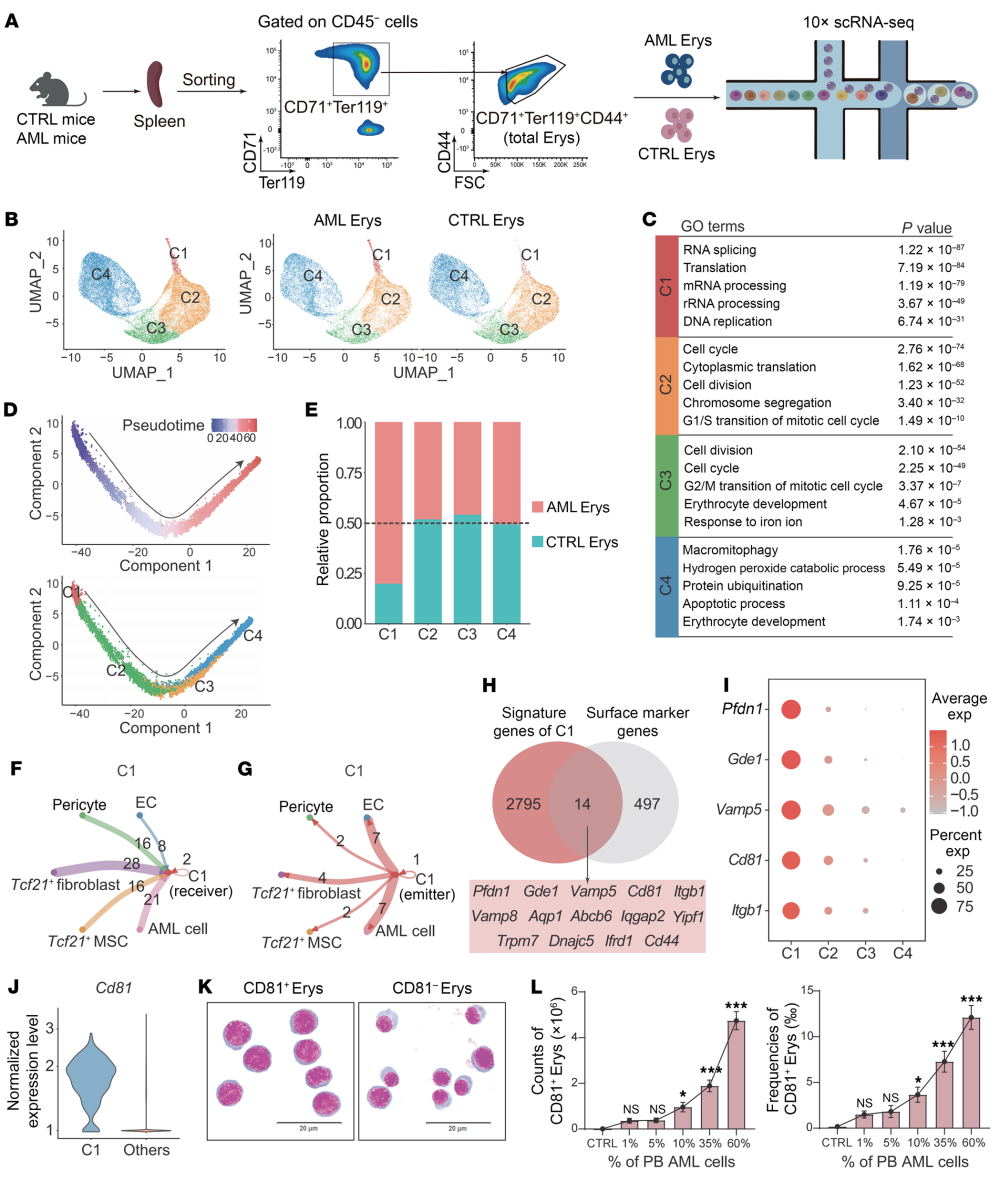
CD81^+^ Erys are notably enriched in the spleens of AML mice. (**A**) A schematic illustrating the experimental design: CD45^−^CD71^+^TER119^+^CD44^+^ erythroblasts (total Erys) from spleens of CTRL mice or mice with advanced AML were sorted by flow cytometry and processed for scRNA-Seq on the 10x Genomics platform. (**B**) UMAP plot showing the cell cluster distribution for AML Erys and CTRL Erys. (**C**) Top 5 gene ontology terms and their corresponding *P* values in the identified clusters. (**D**) Pseudotime analysis illustrating the differentiation trajectory of C1–C4 (top) and the distribution of each cluster (bottom). (**E**) Relative proportions of AML Erys (red) and CTRL Erys (blue) in each cluster. (**F** and **G**) Directional network plots showing the number of incoming (**F**) and outgoing (**G**) interactions for AML C1 Erys and other cells in the AML microenvironment, including AML cells, endothelial cells (ECs), pericytes, *Tcf21*^+^ fibroblasts, and *Tcf21^+^* mesenchymal stem cells (MSCs). (**H**) Venn diagram illustrating the intersection of C1 signature genes and a list of surface marker genes (Supplemental Table 5). The 14 identified genes are listed in the box below the Venn diagram. (**I**) Expression levels of the top 5 C1 surface marker genes across C1–C4. (**J**) Expression of *Cd81* in C1 versus in C2–C4. (**K**) Representative Wright-Giemsa staining of the sorted CD81^+^ Erys and CD81^−^ Erys from the spleens of AML mice. Scale bar: 20 μm. (**L**) Cell counts and frequencies of CD81^+^ Erys in the spleens of CTRL mice and mice at different stages of AML (*n* = 4). Data are presented as the mean ± SEM. The results are representative of 1 of 3 independent experiments with consistent trends. *P < 0.05 and ***P < 0.001, by 1-way ANOVA (**L**).

Prior studies by our group and others have revealed the heterogeneity of erythroblasts (16, 26). To determine whether heterogeneous erythroblast subpopulations exist in the spleens of AML mice and identify which subpopulations mainly contribute to AML progression, we performed single-cell RNA-Seq (scRNA-Seq) on CD45^–^CD71^+^TER119^+^CD44^+^ erythroblasts (total Erys) isolated from the spleens of CTRL mice and advanced-stage AML mice using the 10x Genomics platform (Figure 2A). After implementing rigorous quality control, we integrated the gene expression profiles of the AML and CTRL datasets and obtained 4 major cell clusters (C1–C4) (Figure 2B and Supplemental Table 1–4). We found that erythroid lineage–specific genes (e.g., *Tfrc*, *Gypa*, *Hba-a1*, *Hbb-bt*) were highly expressed across all clusters in both AML and CTRL Erys (Supplemental Figure 2A). Gene ontology analysis indicated that upregulated genes in C1 were associated with RNA splicing, RNA processing, and DNA replication (e.g., *Ybx1*, *Ppia*, *Car1*); genes upregulated in C2 and C3 were linked to active cell cycle, cell division, and chromosome segregation (e.g., *Stmn1*, *Pcna*, *Slbp, Aspm*, *Hmmr*, *Ccnb1*); and genes enriched in C4 were related to protein ubiquitination, apoptosis, and erythrocyte development (e.g., *Xpo7*, *Lpin2*, *Bcl2l1*) (Figure 2C and Supplemental Figure 2, B and C). Trajectory analysis positioned C1 cells at the onset of the erythroid maturation continuum, which extended through C1 to C4 (Figure 2D). Pairwise cluster comparison between AML Erys and CTRL Erys revealed that AML Erys were less mature than their CTRL counterparts, as evidenced by the lower expression of genes involved in heme metabolism and erythrocyte development in AML Erys, such as *Gypa* and *Alas2* (Supplemental Figure 2, D–F).

We next analyzed the distributions of AML Erys and CTRL Erys across C1–C4. Interestingly, AML Erys constituted approximately 80% of C1, whereas C2–C4 contained roughly equal proportions of AML Erys and CTRL Erys, implying that C1 might represent an AML-expanded erythroblast subset (Figure 2E).

To elucidate the potential activities of AML-expanded C1 Erys, we performed an interaction analysis using scRNA-Seq datasets derived from AML Erys and various other cells in the AML splenic microenvironment. Specifically, we conducted scRNA-Seq analyses on AML cells and CD45^−^Ter119^−^CD71^−^ splenic stromal cells isolated from both CTRL mice and advanced AML mice (Supplemental Figure 3A). Unsupervised clustering identified 4 cell populations based on their canonical marker gene expression: endothelial cells (ECs) (*Pecam1*^hi^ and *Cdh5*^hi^, pericytes (*Myh11*^hi^ and *Acta2*^hi^), *Tcf21*^+^ fibroblasts (*Pdgfra*^hi^ and *Dcn*^hi^), and *Tcf21^+^* MSCs (*Csf1*^hi^ and *Cxcl12*^hi^) (Supplemental Figure 3B). Subsequent interaction analysis of erythroblasts and the identified cell subpopulations in their corresponding splenic microenvironment revealed that, compared with AML C2–C4 Erys, AML C1 Erys not only received more frequent and stronger signals from microenvironmental cells, but also emitted more active and intense signals. Notably, AML C1 Erys showed active interaction with AML cells (Figure 2, F and G, and Supplemental Figure 3, C–G). These results collectively suggest that AML-expanded C1 Erys play a key role in AML progression by directly interacting with AML cells and remodeling the splenic microenvironment through active, bidirectional interactions with surrounding cells.

To determine the function of AML-expanded C1 Erys, we next intersected the signature genes of C1 with a curated surface marker gene list obtained from The Human Protein Atlas (31) (Supplemental Table 5). Among the 14 identified genes, *Cd81* was selected for further analysis (Figure 2, H–J). Flow cytometry analysis indicated amplified CD71^+^Ter119^+^CD44^+^CD81^+^ erythroblasts (CD81^+^ Erys) in spleens of AML mice (Supplemental Figure 4, A and B). Analysis of forward scatter (a parameter related to cell size) and the levels of CD71, Ter119, and CD44 — established markers of erythroid maturation (29) — revealed that the majority of CD81^+^ Erys were at early stages of terminal erythropoiesis, predominantly at the proerythroblast and basophilic erythroblast stages (Supplemental Figure 4, C–F), rather than erythroid progenitor stages (Supplemental Figure 4G). This observation was further supported by correlation analysis comparing the transcriptional profiles of clusters C1–C4 with those of proerythroblast, basophilic erythroblast, polychromatic erythroblast, and orthochromatic erythroblast (29) (Supplemental Figure 4H). In accordance, morphological analysis showed that CD81^+^ Erys were larger, had a higher nucleus-to-cytoplasm ratio, and exhibited a more basophilic cytoplasm than CD71^+^Ter119^+^CD44^+^CD81^−^ erythroblasts (CD81^−^ Erys) (Figure 2K and Supplemental Figure 4, I and J). Moreover, the cell counts and frequencies of CD81^+^ Erys increased during AML progression (Figure 2L). Intriguingly, the expansion of CD81^+^ Erys was more evident in the spleens of AML mice than in those of PV, EPO-treated, and PHZ-treated mouse models (Supplemental Figure 4K). Collectively, our findings identify AML-expanded CD81^+^ Erys and reveal that their expansion is specific to AML, suggesting that this cell population may be associated with AML progression.

### CD81^+^ Erys promote AML cell proliferation and contribute to disease progression.

**Figure 3:**
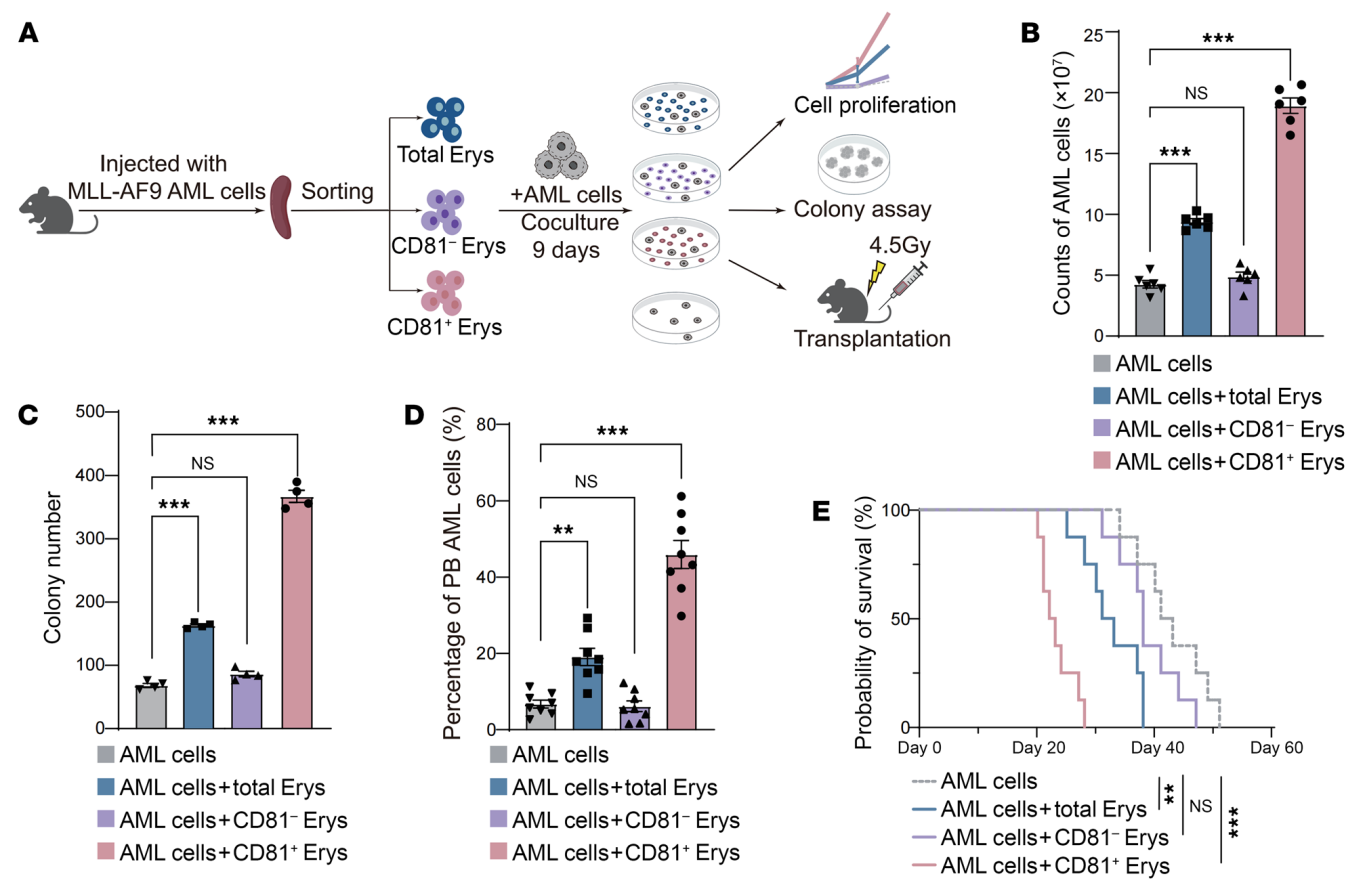
CD81^+^ Erys promote AML cell proliferation and contribute to disease progression. (**A**) A schematic illustrating the experimental design: AML cells were cocultured with total Erys, CD81^−^ Erys, or CD81^+^ Erys isolated from the spleens of mice with advanced AML, or cultured alone, for 9 days. AML cell proliferation in each group was monitored, and cells were then collected for colony formation assays or injection into irradiated (4.5 Gy) mice. (**B**) Cell counts of AML cells obtained after 9 days of culture alone or coculture (*n* = 6). (**C**) Number of colonies formed by cultured AML cells (*n* = 4). (**D** and **E**) AML progression rates determined by the percentage of AML cells in mouse PB (**D**) and survival analysis (**E**) of mice i.v. injected with cultured AML cells (*n* = 8). Data are presented as the mean ± SEM. The results shown are representative of 1 of 3 independent experiments with consistent trends. **P < 0.01 and ***P < 0.001, by 1-way ANOVA (**B**–**D**) and log-rank test (**E**).

To determine the roles of CD81^+^ Erys in AML progression, we conducted in vitro coculture assays. Three erythroblast populations — total Erys, CD81^−^ Erys, and CD81^+^ Erys — were sorted from the spleens of AML mice and cocultured with highly purified AML cells for 9 days (Figure 3A and Supplemental Figure 5, A and B). AML cells cocultured with CD81^+^ Erys showed progressively enhanced proliferation on days 3, 6, and 9 (Supplemental Figure 5C). These cocultured AML cells were evaluated in vitro for proliferation and colony formation assays and in vivo for transplantation assays. We found that CD81^+^ Erys significantly promoted AML cell proliferation, whereas total Erys and CD81^−^ Erys had a moderate and negligible effect, respectively (Figure 3B and Supplemental Figure 5D). This trend was further supported by the colony formation assay (Figure 3C and Supplemental Figure 5E). Remarkably, the AML cells cocultured with CD81^+^ Erys accelerated AML progression after being injected into recipient mice. On day 18 after transplantation, the AML cells from the CD81^+^ Erys coculture group infiltrated approximately 50% of the PB cell population, compared with only around 10% in the AML only or CD81^−^ Erys coculture groups (Figure 3D and Supplemental Figure 5F). Consequently, mice receiving AML cells cocultured with CD81^+^ Erys had a shorter lifespan than those in the other groups (Figure 3E). In summary, our data demonstrate that AML-expanded CD81^+^ Erys could promote AML cell proliferation and accelerate disease progression.

### CD81^+^ Erys secrete elevated levels of macrophage migration inhibitory factor.

**Figure 4:**
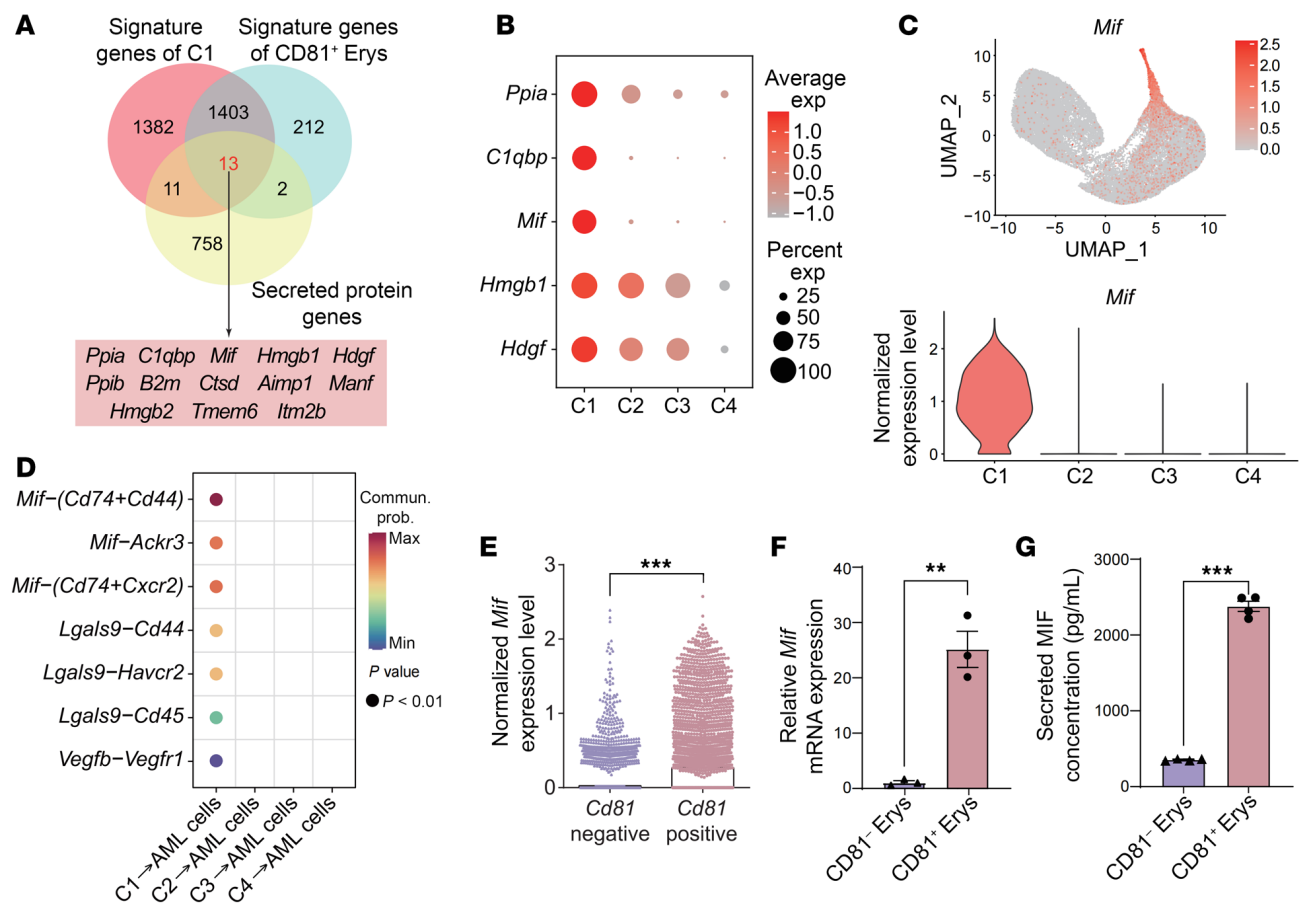
CD81^+^ Erys secrete elevated levels of MIF. (**A**) Venn diagram illustrating the intersection of the C1 and CD81^+^ Erys signature genes with a list of secreted protein genes (Supplemental Table 6). The 13 identified genes are listed in the box below the Venn diagram. (**B**) Expression distributions of the top 5 secreted protein genes across C1–C4. (**C**) Expression of *Mif* across C1–C4. (**D**) Ligand-receptor pairs illustrating the signals emitted by AML C1–C4 Erys and received by AML cells (*P* < 0.01). (**E**) Expression of *Mif* in *Cd81*-negative Erys (relative *Cd81* expression = 0) and *Cd81*-positive Erys (relative *Cd81* expression >1), with the column representing the average expression level (0.02 vs. 0.23). (**F**) Relative RNA expression level of *Mif* in CD81^−^ Erys and CD81^+^ Erys, analyzed by RT-qPCR (*n* = 3). (**G**) Secreted MIF concentration in the culture medium of CD81^−^ Erys and CD81^+^ Erys, measured by ELISA after 12 hours of culture (*n* = 4). Data represent the mean ± SEM. Results are representative of 1 of 3 independent experiments with consistent trends. **P < 0.01 and ***P < 0.001, by 2-tailed, unpaired *t* test (**E**–**G**).

To investigate whether CD81^+^ Erys regulate AML cell proliferation and disease progression by cytokine secretion, we compared the signature genes of C1, the genes upregulated in CD81^+^ Erys compared with CD81^−^ Erys, and a secreted protein gene set obtained from The Human Protein Atlas. This analysis yielded 13 potential candidates (31) (Figure 4A and Supplemental Table 6). Macrophage migration inhibitory factor (*Mif*), which has been reported to boost AML cell growth, was highly expressed in C1 (32) (Figure 4, B and C). Moreover, the cell-cell interaction analysis further indicated *Mif* mediated the strongest interactions between C1 and AML cells (Figure 4D). In accordance with the scRNA-Seq analysis, the isolated CD81^+^ Erys from the spleens of AML mice showed higher MIF expression compared with CD81^−^ Erys (Figure 4, E–G). Considering the previously documented roles of MIF in AML progression (32), our findings raise the intriguing possibility that CD81^+^ Erys might promote AML development through the secretion of MIF.

### MIF plays a central role in mediating the AML-promoting effects of CD81^+^ Erys.

**Figure 5:**
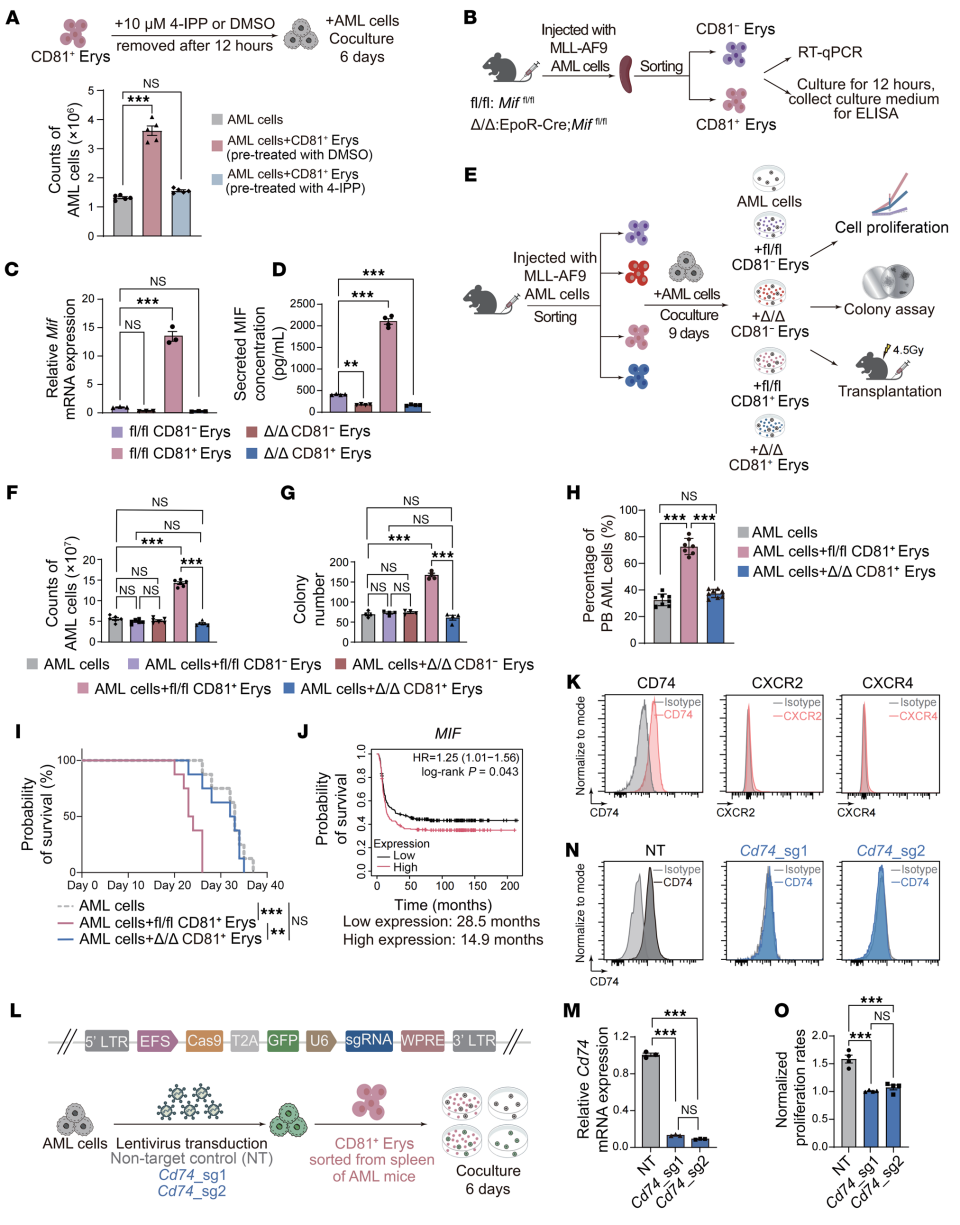
MIF plays a central role in mediating the AML-promoting effects of CD81^+^ Erys. (**A**) Illustration of experimental design (top) and cell counts (bottom) of AML cells cocultured with CD81^+^ Erys pretreated with either 10 μM 4-IPP or DMSO (*n* = 5). (**B**–**D**) Illustration of experimental design (**B**) for AML mouse model induction, flow cytometric sorting of CD81^−^ Erys and CD81^+^ Erys from spleens of *Mif^fl/fl^* (fl/fl) and EpoR-Cre;*Mif^fl/fl^* (Δ/Δ) mice, and confirmation of *Mif* depletion by RT-qPCR (*n* = 3) (**C**) and ELISA (*n* = 4) (**D**). (**E**) Illustration of experimental design: AML cells were cocultured with fl/fl CD81^−^ Erys, Δ/Δ CD81^−^ Erys, fl/fl CD81^+^ Erys, or Δ/Δ CD81^+^ Erys, or cultured alone, for 9 days. AML cell proliferation in each group was monitored; then cells were collected for colony formation assays or injection into irradiated (4.5 Gy) mice. (**F**) Cell counts of AML cells obtained after 9 days of culture alone or coculture (*n* = 6). (**G**) Number of colonies formed by cultured AML cells (*n* = 4). (**H** and **I**) AML progression rates (determined by percentage of AML cells in mouse PB) (**H**) and survival analysis (**I**) of mice injected with cultured AML cells (*n* = 8). (**J**) Correlation between *MIF* expression levels and disease outcomes in AML patient cohorts (GSE1159 and GSE6891; cutoff = median; figure generated using Kaplan-Meier Plotter; ref. 73). (**K**) AML cells were assessed for the expression of CD74, CXCR2, and CXCR4 by flow cytometry. (**L**–**O**) Illustration of construction of nontarget control (NT) and *Cd74*_KO (*Cd74*_sg1, *Cd74*_sg2) AML cells, followed by measurement of CD74 expression by RT-qPCR (*n* = 3) (**M**) and flow cytometry (**N**), and assessment of proliferation rates of NT and *Cd74*_KO AML cells cocultured with or without CD81^+^ Erys (*n* = 4) (**O**). Rates were calculated as the ratio of AML cell counts obtained after coculturing with CD81^+^ Erys to those obtained after culture alone. Data represent the mean ± SEM. Results are representative of 1 of 3 independent experiments with consistent trends. **P < 0.01 and ***P < 0.001, by 1-way ANOVA (**A**, **C**, **D**, **F**, **G**, **H**, **M**, **O**) and log-rank test (**I** and **J**).

To determine whether MIF mediated the AML-promoting effects of CD81^+^ Erys, we pretreated CD81^+^ Erys with the MIF-specific inhibitor 4-iodo-6-phenylpyrimidine (4-IPP), which irreversibly inhibits the biological activity of MIF (33), and cocultured the 4-IPP–pretreated CD81^+^ Erys with AML cells. The results showed that pretreatment with 4-IPP significantly inhibited the pro-proliferative effects of CD81^+^ Erys on AML cell proliferation, confirming MIF’s essential role in this process (Figure 5A).

To further elucidate the functional importance of CD81^+^ Erys-derived MIF in AML progression, we generated EpoR-Cre;*Mif^fl/fl^* (Δ/Δ) mice, in which the *Mif* gene was primarily deleted in the erythroid lineage, as well as in other hematopoietic lineages (Supplemental Figure 6, A and B) (34). Peripheral hemogram parameters, splenic size, and erythroid differentiation (in the BM and spleen) were comparable between Δ/Δ mice and their *Mif^fl/fl^* (hereafter, fl/fl mice) littermates, suggesting that *Mif* deletion did not perturb normal erythropoiesis (Supplemental Figure 6, C–K).

After AML induction, total Erys isolated from the spleens of Δ/Δ mice showed significantly reduced MIF levels compared with those from fl/fl mice (Supplemental Figure 6, L–N). Specifically, *Mif* expression was markedly lower in Δ/Δ CD81^+^ Erys compared with fl/fl CD81^+^ Erys (Figure 5, B–D). We then isolated CD81^+^ Erys and CD81^−^ Erys from the spleens of Δ/Δ and fl/fl mice with advanced AML and cocultured them with AML cells for 9 days (Figure 5E). AML cells cocultured with fl/fl CD81^+^ Erys exhibited higher proliferative capacity than that of AML cells cocultured with Δ/Δ CD81^+^ Erys (Figure 5F and Supplemental Figure 6O). Colony formation assays further confirmed the indispensable role of MIF in promoting AML cell proliferation (Figure 5G and Supplemental Figure 6P). Importantly, mice injected with AML cells that were cocultured with Δ/Δ CD81^+^ Erys exhibited significantly slower AML progression and longer survival compared with those injected with AML cells cocultured with fl/fl CD81^+^ Erys (Figure 5, H and I, and Supplemental Figure 6Q).

Consistent with our findings, clinical data showed that elevated *MIF* expression was correlated with poor survival outcomes in patients with AML (Figure 5J). Together, our data reveal that CD81^+^ Erys in the spleens of AML mice promote AML cell proliferation and disease progression primarily through MIF secretion.

### MIF promotes AML cell proliferation through binding to CD74 on AML cells.

MIF exerts its biological effects through various receptors, including CD74, CXCR2, and CXCR4. To determine which receptors were implicated in MIF signaling in AML, we assessed their expression on AML cells. We found that CD74 was highly expressed, whereas CXCR2 and CXCR4 levels were negligible (Figure 5K). This finding aligned with the results of cell communication analysis, which suggested that MIF secreted by C1 exerted its effects on AML cells via CD74 (Figure 4D).

To validate the role of MIF/CD74 interaction in AML cell proliferation, we generated *Cd74*_KO AML cell lines (Figure 5, L–N). Importantly, the coculture effect induced by CD81^+^ Erys was attenuated in *Cd74*_KO AML cells compared with the nontarget control (NT) (Figure 5O). Thus, our results suggest that the MIF/CD74 axis mediates the effects of CD81^+^ Erys on AML cell proliferation.

### Coculturing with CD81^+^ Erys reshapes the metabolic profile of AML cells.

To investigate the molecular mechanisms driving AML proliferation in coculture with CD81^+^ Erys, we performed bulk RNA-Seq on AML cells (purity >99%) cultured alone or cocultured with CD81^+^ Erys or CD81^–^Erys (Supplemental Figure 7A).

**Figure 6:**
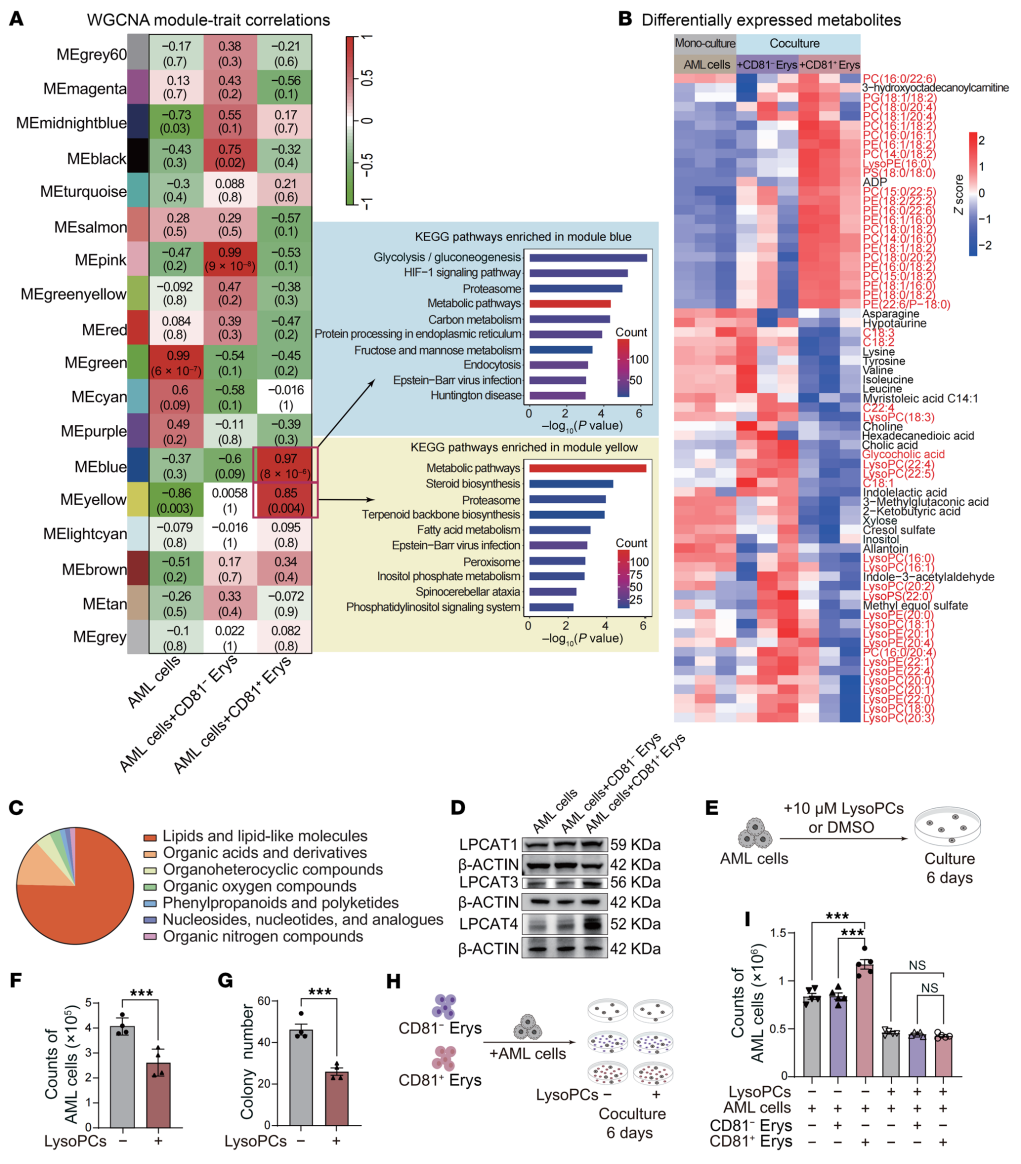
Coculturing with CD81^+^ Erys reshapes the metabolic profile of AML cells. (**A**) Module-trait correlations derived from weighted gene coexpression network analysis (WGCNA) of all expressed genes in AML cells that were either cultured alone or cocultured with CD81^−^ Erys or CD81^+^ Erys and subsequently sequenced. KEGG analysis of genes in the blue and yellow modules was performed to identify the most significantly enriched pathways. The module eigengenes (ME) represent the overall gene expression profile. (**B**) Identification of metabolites specifically altered in AML cells cocultured with CD81^+^ Erys. Differential abundance was determined by comparing both CD81^+^ and CD81^−^ Erys cocultures with AML monoculture. Selection criteria were set at a raw *P* value less than 0.05 and an FDR less than 0.2 for both comparisons. (**C**) Classification of differentially expressed metabolites identified in AML cells cocultured with CD81^+^ Erys using the ClassyFire superclass annotation. (**D**) Protein expression levels of LPCAT1, LPCAT3, and LPCAT4 in AML cells cultured alone or cocultured with CD81^−^ Erys or CD81^+^ Erys. β-Actin was used as a loading control. Molecular weights are indicated on the right. (**E**) A schematic illustrating the experimental design for lysophosphatidylcholine (LysoPC) supplementation in AML monoculture. (**F**) Cell counts of AML cells obtained after 6 days of culture (*n* = 4). (**G**) Number of colonies formed by cultured AML cells (*n* = 4). (**H**) A schematic illustrating the experimental design: AML cells were cocultured with CD81^−^ Erys or CD81^+^ Erys, or cultured alone, for 6 days, with or without LysoPC supplementation. (**I**) Cell counts of AML cells obtained after 6 days of culture (*n* = 5). Data are presented as the mean ± SEM. The results shown are representative of 1 of 3 independent experiments with consistent trends. ***P < 0.001, by 2-tailed, unpaired *t* test (**F** and **G**) and 1-way ANOVA (**I**).

Using weighted gene coexpression network analysis (WGCNA), we identified 2 gene modules (the blue and yellow modules, identified based on a module-trait correlation greater than 0.85 and a *P* value less than 0.05, Figure 6A) specifically enriched in AML cells cocultured with CD81^+^ Erys. Kyoto Encyclopedia of Genes and Genomes (KEGG) analysis revealed these modules were associated with glycolysis/carbon metabolism and steroid biosynthesis/fatty acid metabolism (Figure 6A and Supplemental Tables 7–10), suggesting that CD81^+^ Erys may drive metabolic disturbances in AML cells through coculture.

Metabolomic profiling showed that over 75% of the differential metabolites in AML cells cocultured with CD81^+^ Erys were lipids and lipid-like metabolites. This included elevated phospholipids, such as phosphatidylcholines, and phosphatidylethanolamines, alongside a marked reduction in lysophospholipids, particularly lysophosphatidylcholines (LysoPCs) (Figure 6, B and C). LysoPCs are of particular interest given previous reports that their accumulation can suppress tumor progression (35, 36). Correspondingly, we observed an upregulation in the protein expression of lysophospholipid acyltransferases, including LPCAT1, LPCAT3, and LPCAT4 (Figure 6D), which catalyze the reacylation of lysophospholipids to phospholipids in AML cells cocultured with CD81^+^ Erys. Since ROS can promote lipid peroxidation and LysoPC generation (37), we quantified intracellular ROS but found no significant differences among AML cells cultured alone or cocultured with CD81^+^ or CD81^–^ Erys (Supplemental Figure 7B). These results suggest that LysoPC reduction in AML cells cocultured with CD81^+^ Erys occurs independently of ROS-mediated lipid peroxidation.

To determine the consequence of unbalanced phospholipid metabolism, particularly the role of LysoPCs in AML cells, we investigated the effect of LysoPC supplementation in AML monoculture (Figure 6E). Exogenous supplementation significantly inhibited both the proliferation and colony formation of AML cells (Figure 6, F and G, and Supplemental Figure 7C). Inspired by this observation, we next added LysoPCs to the AML-erythroblast coculture system (Figure 6H) and found that the coculture effect induced by CD81^+^ Erys was nearly completely abrogated (Figure 6I and Supplemental Figure 7D). This result suggests that metabolic remodeling contributes to the enhanced proliferation of AML cells during coculture with CD81^+^ Erys.

### MIF/CD74/mTORC1/EGLN3 regulatory axis rebalances lipid metabolism in AML cells during coculture.

**Figure 7:**
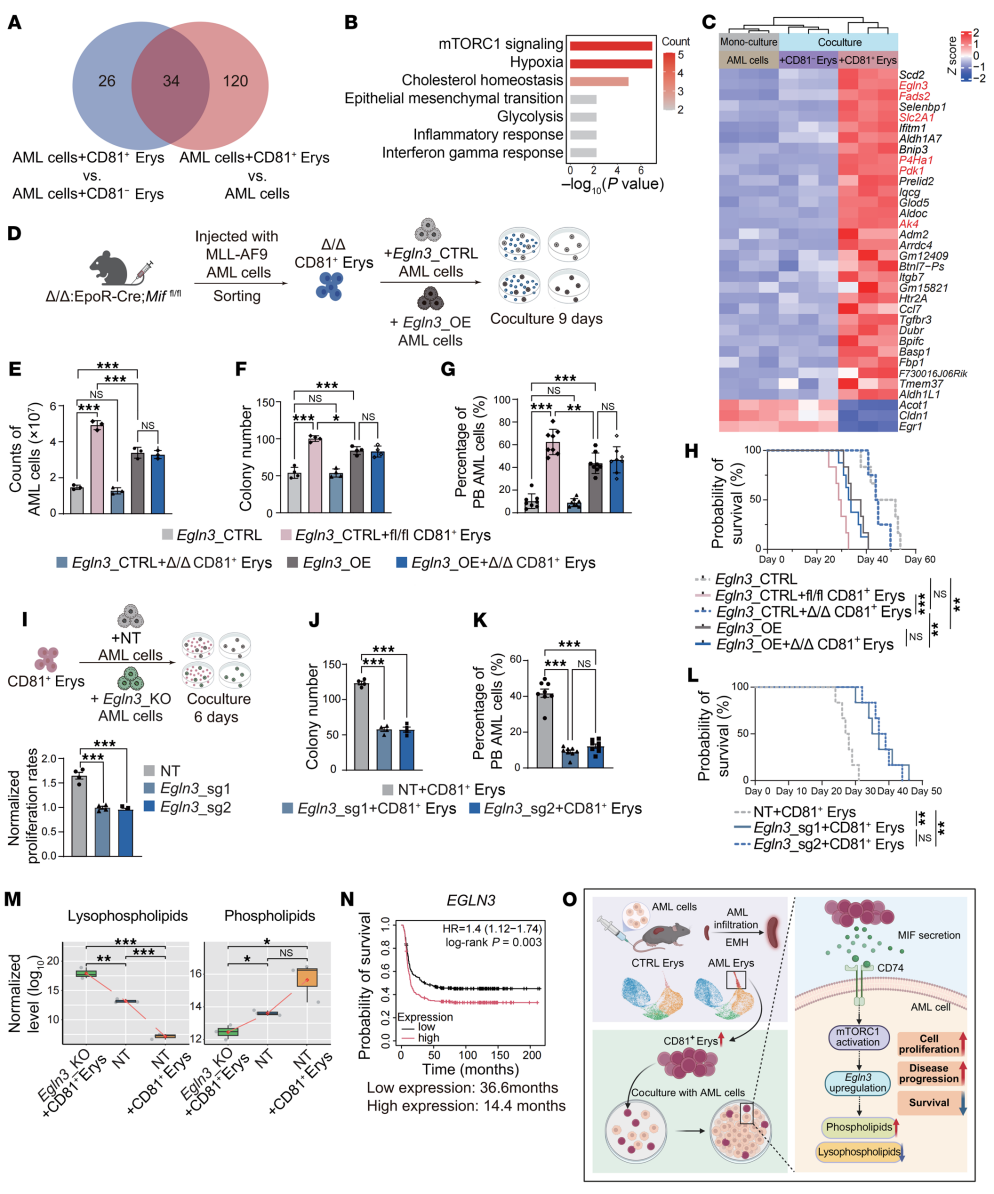
MIF/CD74/mTORC1/EGLN3 regulatory axis rebalances lipid metabolism in AML cells during coculture. (**A**) Venn diagram illustrates selection of 34 DEGs from AML cells after coculture with CD81^+^ Erys. DEGs were identified by intersecting those upregulated in AML cells cocultured with CD81^+^ Erys versus AML cells cocultured with CD81^−^ Erys (AML cells+CD81^+^ Erys vs. AML cells+CD81^−^ Erys) and compared with AML cells alone (AML cells+CD81^+^ Erys vs. AML cells); *P*_adj_ < 0.05. (**B**) Hallmark overrepresentation analysis of 34 DEGs. (**C**) Expression of 34 DEGs, including 6 core enrichment genes of mTORC1 signaling pathway (in red). (**D**) Illustration of experimental design: *Egln3*_CTRL or *Egln3*_OE AML cells were cultured alone or cocultured with Δ/Δ CD81^+^ Erys for 9 days. (**E**) Cell counts of AML cells obtained after 9 days of culture alone or coculture (*n* = 3). *Egln3_CTRL* AML cells cocultured with fl/fl CD81^+^ Erys were positive control. (**F**) Number of colonies formed by cultured AML cells (*n* = 4). (**G** and **H**) AML progression rates (determined by percentage of AML cells in mouse PB) (**G**) and survival analysis (**H**) of mice injected with cultured AML cells (*n* = 8). (**I**) Experimental design for coculturing nontarget control (NT) and *Egln3*_KO (*Egln3*_sg1, *Egln3*_sg2) AML cells with CD81^+^ Erys (top). Proliferation rates of NT and *Egln3*_KO AML cells were assessed after 6 days of culture alone or coculture with CD81^+^ Erys (*n* = 4) (bottom). Rates were calculated as the ratio of AML cell counts obtained after coculturing with CD81^+^ Erys to those obtained after culture alone. (**J**) Number of colonies formed by cultured AML cells (*n* = 4). (**K** and **L**) AML progression rates (determined by percentage of AML cells in mouse PB) (**K**) and survival analysis (**L**) of mice injected with cultured AML cells (*n* = 8). (**M**) Relative abundance of lysophospholipids and phospholipids in AML cells under different experimental conditions (*n* = 3). (**N**) Correlation between *EGLN3* expression levels and disease outcomes in AML patient cohorts (GSE1159 and GSE6891; cutoff = median; figure generated using Kaplan-Meier Plotter; ref. 73). (**O**) Left: Infiltration of AML cells into spleen drives EMH and expansion of CD81^+^ Erys, fueling disease progression. Right: Molecular mechanism by which MIF, secreted by CD81^+^ Erys, interacts with CD74 on AML cells, activating mTORC1 pathway and upregulating downstream *Egln3*. This signaling cascade disrupts the metabolic balance between phospholipids and lysophospholipids, creating a favorable environment that supports AML cell proliferation, survival, and disease progression. Data are shown as the mean ± SEM. Results are representative of 1 of 3 independent experiments with consistent trends. *P < 0.05, **P < 0.01, and ***P < 0.001, by 1-way ANOVA (**E**–**G**, **I**–**K**, and **M**) or log-rank test (**H**, **L**, **N**).

To further explore the molecular mechanisms underlying the dysregulated lipid profile and their coculture effects caused by CD81^+^ Erys coculture, we intersected the genes upregulated in AML cells under 2 comparison sets: (a) cocultured with CD81^+^ Erys versus CD81^−^ Erys, (b) cocultured with CD81^+^ Erys versus cultured alone. This led to the identification of 34 differentially expressed genes (DEGs) (Figure 7A and Supplemental Tables 11 and 12). HALLMARK overrepresentation analysis revealed that these enriched DEGs were implicated in mTORC1 signaling, hypoxia response, and cholesterol homeostasis (Figure 7B). Among them, the mTORC1 signaling pathway was markedly elevated in AML cells cocultured with CD81^+^ Erys (Supplemental Figure 8A). The proliferation of AML cells was significantly inhibited by supplementation with the mTORC1 inhibitor rapamycin (50 nM) into the coculture system (Supplemental Figure 8B). Therefore, it is likely that CD81^+^ Erys modulated AML cell proliferation via the MIF/CD74/mTORC1 regulatory axis. This hypothesis was further supported by the observation that *mTOR* expression was attenuated in AML cells cocultured with Δ/Δ CD81^+^ Erys, indicating that mTORC1 activation depends on MIF secretion from CD81^+^ Erys (Supplemental Figure 8C).

To further pinpoint the key gene in AML cells associated with the mTORC1 signaling pathway upon coculture with CD81^+^ Erys, we identified 34 DEGs and found that *Egln3* (Egl-9 family hypoxia inducible factor 3) was the most significantly upregulated gene downstream of the mTORC1 signaling pathway (Figure 7C). The robust upregulation of *Egln3* in AML cells from the CD81^+^ Erys coculture group was confirmed, and this upregulation was dependent on the MIF/CD74/mTORC1 regulatory axis, as evidenced by the failure of both Δ/Δ CD81^+^ Erys and mTORC1 inhibitor to elevate *Egln3* expression (Supplemental Figure 8, D–F).

To validate *Egln3* as a downstream effector of MIF, we overexpressed *Egln3* (*Egln3*_OE) in AML cells and cocultured them with Δ/Δ CD81^+^ Erys for 9 days (Figure 7D and Supplemental Figure 8G). We found that *Egln3* overexpression partially restored the proliferative advantage of AML cells when cocultured with Δ/Δ CD81^+^ Erys (Figure 7E and Supplemental Figure 8H), consistent with the results of colony formation assays (Figure 7F and Supplemental Figure 8I). Additionally, we transplanted the cocultured *Egln3*_CTRL and *Egln3*_OE AML cells into sublethally irradiated (4.5 Gy) recipient mice. Mice with *Egln3*_OE AML cells showed significantly accelerated leukemia progression and shorter survival (Figure 7, G and H, and Supplemental Figure 8J). These findings collectively demonstrate that *Egln3* overexpression can compensate for MIF deficiency, supporting its role as a downstream effector in the MIF/CD74/mTORC1 signaling axis.

To further strengthen the functional role of *Egln3* in AML proliferation and disease progression, and its potential involvement in metabolic reprogramming, we generated *Egln3*_KO AML cell lines (Supplemental Figure 8, K and L). We found that *Egln3* depletion significantly attenuated the proliferative advantage conferred by CD81^+^ Erys (Figure 7, I and J, and Supplemental Figure 8M). More importantly, mice injected with CD81^+^ Erys-cocultured *Egln3*_KO AML cells exhibited markedly slower disease progression, with fewer PB AML cells (Figure 7K and Supplemental Figure 8N) and longer survival (Figure 7L).

To uncover the underlying mechanisms responsible for the reduced AML cell proliferation, we conducted metabolomic profiling of *Egln3*_KO AML cells and their corresponding NT AML cells after coculture with CD81^+^ Erys. The most notable metabolite alterations after *Egln3* depletion were the opposing trends of lysophospholipids and phospholipids. We found that *Egln3* depletion led to significantly decreased phospholipids, including phosphatidylcholines and phosphatidylethanolamines, and increased lysophospholipids, particularly LysoPCs (Figure 7M and Supplemental Figure 8O). In summary, the metabolomic profile of *Egln3*_KO AML cells cocultured with CD81^+^ Erys exhibited an opposite trend compared with that of NT AML cells cocultured with CD81^+^ Erys, underscoring the critical role of *Egln3* in this metabolic adaptation.

In addition, the beneficial effect of low *EGLN3* expression was further corroborated by a positive correlation between increased *EGLN3* expression and adverse outcomes in patients with AML (Figure 7N). In conclusion, our findings suggest that the impact of CD81^+^ Erys on AML cells is at least partially mediated by *Egln3* (Figure 7O).

## Discussion

Previous studies have elaborated on the important role of the niche, either as a driver or accomplice, in various malignancies (38, 39). However, the full repertoire of niche components and how they interact to enhance tumor survival, particularly during metastasis, are still not completely understood. During leukemia progression, the spleen is not only the primary organ responsible for EMH but also a major organ infiltrated by malignant cells (40). Thus, AML cells, non-AML hematopoietic cells, and other microenvironmental cells coexist in the spleen, forming an ideal model for studying the niche during tumor metastasis. For a long time, most niche investigations have been restricted to CD45^+^ classic immune cells, including myeloid-derived suppressor cells (MDSCs), T cells, and NK cells, or CD45^–^ nonhematopoietic stromal cells like MSCs, ECs, and fibroblasts in the BM (1–5). Here, we reveal that erythroblasts account for the highest proportion of nonleukemic hematopoietic cells in the spleen during EMH and constitute an essential component of the tumor microenvironment in AML. It is well established that during tumor progression, malignant cells actively interact with their microenvironment to reshape it in their favor (41–43). In this study, we found that AML cell infiltration in the spleen induced the expansion of CD81^+^ Erys, which actively interacted with various microenvironmental cells to create a permissive niche that fuels disease progression in a self-reinforcing manner.

We have demonstrated that AML-expanded CD81^+^ Erys cells have functions beyond solely serving as ancestral cells required for mature RBC differentiation. In accordance with our findings, noncanonical functions of nucleated erythroid cells have been reported in various cancer contexts. For example, CD45^−^Ter119^+^CD71^+^ erythroblast-like cells in the spleens of patients with hepatocellular carcinoma and in a corresponding disease mouse model were shown to facilitate cancer cell metastasis via the secretion of artemin (24, 25). Additionally, splenic erythroid progenitors (CD45^+^CD71^+^Ter119^+^) confer potent immunosuppressive effects in mice with lung cancer by inhibiting CD8^+^ T cells (22) or by transdifferentiating into erythroid-derived myeloid cells (23). Because of the critical physiological functions of erythroid cells, their complete eradication during cancer treatment is not feasible; however, analyzing the heterogeneity in erythroid cells offers a strategic approach for the selective elimination of detrimental RBC subsets while preserving the integrity of beneficial ones. Indeed, recent research has revealed that erythroid cells might not be as homogenous as previously thought. For instance, a CD63^+^ immune-prone erythroblast subpopulation is formed during steady-state erythropoiesis and is maintained throughout human ontogeny (26). Moreover, a VISTA^+^ erythroblast subpopulation with immunomodulatory properties has been identified in neonatal mice (16). In the present study, we identified a CD81^+^ Erys subset that facilitated AML progression. With a growing appreciation for erythroid heterogeneity, future studies may reveal how erythroid cells contribute to various biological processes.

We demonstrated that CD81^+^ Erys are capable of reprogramming the metabolism of AML cells, specifically by disturbing the balance between phospholipids and lysophospholipids to facilitate AML amplification. Given that LysoPCs have been reported to inhibit cancer cell proliferation and migration, their reduction in CD81^+^ Erys coculture conditions may represent an adaptive mechanism that favors AML progression (35, 44). These findings suggest that targeting the metabolic crosstalk between CD81^+^ Erys and AML cells could provide a potential therapeutic strategy for disrupting the tumor-supportive microenvironment in AML.

We demonstrated that the MIF/CD74/mTORC1/EGLN3 regulatory axis underlies the enhanced proliferation of AML cells conferred by CD81^+^ Erys. Previous studies have shown that the MIF/CD74 interaction modulates mTORC1 signaling and induces hypoxia (45–49), with EGLN3 acting as a key downstream mediator. By mediating the degradation of HIF subunits, EGLNs serve as crucial regulators of the hypoxic response (50). Based on the findings that *Hif* depletion accelerates AML disease progression in mice (51–53) and inhibition of other EGLN family members efficiently compromises AML development (54), we hypothesized that *Egln3* upregulation in AML cells could promote AML progression. Furthermore, EGLN3 actively participates in various physiological and pathological processes (55), including the activation of glycolysis (56), mitochondrial biogenesis (57), and cell cycle progression (58). Here, we further establish a connection between *Egln3* in AML cells cocultured with CD81^+^ Erys and lipid metabolic reprogramming, specifically the balance between phospholipids and lysophospholipids. Interestingly, *Egln3* may exert distinct roles under different metabolic conditions. In AML cells cultured alone, *Egln3* is associated with fatty acid oxidation, playing a key role in regulating cell metabolism and fitness (59). In contrast, upon coculture with CD81^+^ Erys and accompanying metabolic remodeling, the function of *Egln3* may shift to regulate the conversion between phospholipids and lysophospholipids. Nonetheless, the metabolic regulatory role of *Egln3* awaits further investigation.

In summary, our findings reveal that CD81^+^ Erys play a pivotal role in remodeling the AML microenvironment to promote disease progression. The metabolic and molecular crosstalk between CD81^+^ Erys and AML cells highlights the unique capacity of erythroblasts to act as critical regulators within the leukemic microenvironment. Targeting this erythroblast-driven pathway or resultant metabolic alterations offers a promising therapeutic strategy for AML and other hematological malignancies involving EMH.

## Methods

### Sex as a biological variable.

Our study examined male and female animals, and similar findings are reported for both sexes.

### Mice.

C57BL/6J and B6.SJL mice were purchased from the animal facility of the State Key Laboratory of Experimental Hematology. *Mif^fl/+^* mice were generated by Cyagen Biosciences (Suzhou) Inc., and EpoR-tdTomato-Cre mice were provided by Xiuli An (New York Blood Center, Rye, New York, USA). (34). Polycythemia vera mice (*JAK2*^V617F^-transgenic mice, PB HGB >190 g/L) have been previously characterized and documented in a prior study (60); mice were 8–12 weeks of age at the time of experimentation. To induce stress erythropoiesis, 8- to 12-week-old mice were subjected to either (PHZ) (MilliporeSigma, 114715) or EPO (Peprotech, 100-64) treatment. For PHZ treatment, mice received i.p. injections of PHZ (100 mg/kg) on day 0 and were euthanized on day 4. For EPO treatment, mice were i.p. administered EPO (50 U/day) for 5 days, and spleens were collected on day 6.

### Generation of leukemia model.

MLL-AF9 AML cells were generated using a previously established protocol (6). Briefly, Lin^−^c-kit^+^ cells were isolated from the BM of B6.SJL mice and transduced with a retrovirus carrying the *MLL-AF9* fusion gene. These modified cells were then transplanted into 8–12-week-old C57BL/6J mice, which were half-lethally irradiated (4.5 Gy). The recipient mice were monitored until the study endpoint, and both BM and spleen cells were carefully collected and stored in liquid nitrogen.

To establish the nonirradiated AML mouse model, AML cells obtained from C57BL/6J mice were injected via the tail vein into secondary, nonirradiated recipients. The progression of AML in these recipients was monitored by tracking the proportion of AML cells in their PB.

### Flow cytometry.

Cells were obtained by gently flushing the BM or grinding the spleen, then suspended in PBS containing 10% FBS (vol/vol). The single-cell suspension was passed through a 70 μm cell strainer to remove debris (BD Falcon). The following antibodies were used for analyzing leukocytes: anti–CD45.1-FITC (BioLegend, 110705), anti–CD45.2-BV510 (BioLegend, 109837), anti–CD4-APC (BioLegend, 100412), anti–CD8-APC/Cy7 (BioLegend, 100714), anti–B220-PerCp/Cy5.5 (BioLegend, 103234), anti–Gr1-PE/Cy7 (BioLegend, 108416), anti–CD11b-PE/Cy7 (BioLegend, 101216), and anti–F4/80-PE (BioLegend, 111603). The following antibodies were used for analyzing erythroblasts: anti–CD45.1-FITC (BioLegend, 110705), anti–CD45.2-BV510 (BioLegend, 109837), anti–CD71-PE (BioLegend, 113808), anti–Ter119-APC (BioLegend, 116212), anti–CD44-PerCp/Cy5.5 (BioLegend, 103032), anti–CD81-PE/Cy7 (BioLegend, 104914), anti–CD11b-APC/Cy7 (BioLegend, 101226), and anti–Gr1-APC/Cy7 (BioLegend, 108424). The following antibodies were used for analyzing erythroid progenitors: anti–CD49f-Percp/Cy5.5 (BioLegend, 313618), anti–CD117-BV605 (BioLegend, 313618), anti–Ter119-eFluor780 (Invitrogen, Thermo Fisher Scientific, 47-5921-82), anti–CD55-PE/Cyanine7 (BioLegend, 131814), anti–CD150-BV421 (BioLegend, 115926), anti–CD105-APC (BioLegend, 120414), anti–CD71-FITC (BioLegend, 113806), and anti–CD45.1-PE (BioLegend, 110708). Samples were collected using the BD FACSCanto II flow cytometer and analyzed using FlowJo software (BD Biosciences).

### Cell sorting.

Single-cell suspensions, prepared as described in *Flow cytometry*, were purified using anti-CD45 microbeads (Miltenyi Biotec, 130-052-301) and anti-PE microbeads (Miltenyi Biotec, 130-105-639); for EpoR-Cre mice, anti-FITC microbeads (Miltenyi Biotec, 130-048-701) were used according to the manufacturer’s instructions to enrich CD45^−^CD71^+^ cells. Briefly, the cells were incubated with anti-CD45 microbeads at 4°C for 30 minutes, washed once, and resuspended in PBE buffer (PBS solution supplemented with the following: 1% penicillin-streptomycin, Gibco, Thermo Fisher Scientific, 15140-122; 2% FBS, Gibco, 16000-044; and 0.4% 0.5 M EDTA solution, Invitrogen, Thermo Fisher Scientific, 15575-038). The cell suspension was then transferred onto an LS column (Miltenyi Biotec, 130-042-401), and the flow-through was collected. Unlabeled cells were stained with anti–CD71-PE antibody at 4°C for 30 minutes, then incubated with anti-PE microbeads under the same conditions (anti–CD71-FITC antibody and anti-FITC microbeads were used for cells from EpoR-Cre mice). After another wash, the cells were resuspended in PBE buffer and loaded onto an LS column. Labeled cells within the column were then collected.

The enriched CD45^−^CD71^+^ cells were labeled with antibodies in the same manner as described for the analysis of erythroblasts. Cellular suspensions were treated with DAPI (1 μg/mL, MilliporeSigma) to enable the exclusion of nonviable cells. Erythroblasts (CD45^−^Gr1^−^CD11b^−^CD71^+^Ter119^+^CD44^+^) were sorted using the BD FACSAria III cell sorter (BD Biosciences).

### Wright-Giemsa staining.

Wright-Giemsa staining (BaSO Biotech; BA4017) of cyto-spin preparations was performed according to the manufacturer’s instructions. Briefly, the cyto-spin preparation was incubated for 30 seconds with 70 μL solution A, followed by a 7-minute incubation with 140 μL of solution B. The stain was then gently washed off with a stream of water.

### In vitro coculture assay.

Two days before erythroblast sorting, MLL-AF9 AML cells were thawed and suspended in PBS. After centrifugation, the cell pellet was resuspended in IMDM (Gibco, Thermo Fisher Scientific, 12440053) supplemented with 15% FBS (Gibco, Thermo Fisher Scientific, 16000-044), 10 ng/mL mouse IL-3 (Peprotech, 213-13), 10 ng/mL mouse IL-6 (Peprotech, 216-16), 50 ng/mL mouse stem cell factor (SCF) (Peprotech, 250-03), and 1% penicillin-streptomycin (Gibco, Thermo Fisher Scientific, 15140-122). The cells were then maintained in an incubator at 5% CO_2_ and 37°C.

In erythroblast–AML coculture assays, we began with a consistent cell seeding strategy, introducing 3,000 AML cells alongside 30,000 Erys in each coculture setup. Every 3 days, we counted the number of AML cells in the Erys coculture group and replenished the culture with a 10-fold excess of fresh Erys across all experimental settings. During the culture period, the following drugs were added to the medium as specified: 4-IPP (MCE, HY-110063), which was used to pretreat CD81^+^ Erys for 12 hours and removed prior to coculture; AKB-6899 (MCE, HY-113649); rapamycin (MCE, HY-10219); and LysoPCs (MCE, HY-139414).

### Colony formation assay and AML cell transplantation into mice.

To assess the colony-forming potential of AML cells, they were purified by sorting after 9 days of culture alone or coculture with erythroblasts. The cells were then seeded in methylcellulose (STEMCELL Technologies, M3231) according to the manufacturer’s guidelines. Additionally, AML cells were i.v. injected into half-lethally irradiated (4.5 Gy) recipient mice to assess their pathogenicity.

### Plasmids, viral production, and cell transduction.

For the *Cd74* and *Egln3* KO assays, sgRNA oligonucleotides were custom-synthesized by Tsingke and subsequently cloned into the pLenti-Cas9-GFP vector (Addgene plasmid, 86145). The list of control and target-specific sgRNA primers used in these experiments is provided in Supplemental Table 13.

For lentivirus production, HEK293T cells with a low passage number were transfected with lentiviral vectors. Briefly, the pSPAX2, pMD2.G, and pLenti-Cas9-GFP plasmids were mixed at a mass ratio of 3:2:1 and transfected into HEK293T cells using Lipofectamine 2000 transfection reagent (Invitrogen, Thermo Fisher Scientific, 11668500). The cell supernatant containing the virus was collected at 48–72 hours after transfection, filtered through a 0.45 μm filter (Pall Corporation, 4614), and stored at –80°C.

For cell transduction, 5 × 10^5^ MLL-AF9 AML cells were seeded onto a vitronectin-coated 24-well plate. The cells were then exposed to lentivirus at an appropriate MOI and 8 μg/mL polybrene (Beyotime, C0351). The cells were centrifuged with the virus at 600*g* and 37°C for 90 minutes. The culture medium was replaced 8 hours after centrifugation, and the transduced cells (GFP^+^) were sorted 48 hours later.

### ELISA.

For ELISA, 1 × 10^5^ erythroblasts from the spleens of AML or CTRL mice were seeded in 100 μL of IMDM supplemented with 10% FBS. After a 12-hour incubation, the cells were centrifuged at 350*g* for 10 minutes, and the resulting supernatant was collected. The concentration of MIF in the culture supernatant was quantified using a commercially available mouse MIF ELISA kit (Proteintech, KE10027).

### Quantitative reverse transcription PCR.

Total cellular RNA was extracted using TRIzol reagent (Invitrogen, Thermo Fisher Scientific, 15596018CN). A 1 μg sample of total RNA was reverse-transcribed in a 20 μL reaction volume using the Hifair III 1st Strand cDNA Synthesis SuperMix (including gDNA digestion) (Yeasen, 11141ES). The quantitative reverse transcription PCR primer sequences are provided in Supplemental Table 14. The reverse transcription products from different samples were then amplified using the QuantStudio 6 Pro Real-Time PCR System (Thermo Fisher Scientific, A43168) with Hieff UNICON Universal Blue qPCR SYBR Green Master Mix (Yeasen, 11184ES), following the manufacturer’s guidelines. The resulting data were normalized to the expression level of the *β-actin* gene.

### scRNA-Seq and data processing.

Spleens from AML and CTRL mice were processed to prepare a single-cell suspension. CD45^−^ cells were enriched, and total erythroblasts (CD45^−^CD11b^−^Gr1^−^CD71^+^Ter119^+^CD44^+^) were isolated by flow cytometry. scRNA-Seq libraries were generated using the 10x Chromium Controller with V2 chemistry, following the manufacturer’s guidelines. These libraries were then sequenced on the Illumina HiSeq X Ten platform at Novogene in Beijing.

The dataset generated through the 10x Genomics platform underwent initial processing, with the raw data in FASTQ format aligned to the GRCm38 mouse reference genome. This alignment process was performed using the Cell Ranger (v3.0.2) pipeline (10x Genomics) with default parameters. A quality control procedure was implemented to assess the data quality from the 10x Genomics platform. First, potential doublets were removed using Scrublet (v0.2.1) (61). Next, data from genes expressed in more than 3 cells and cells expressing more than 500 genes with less than 10% mitochondrial gene content were retained for further analysis.

The Seurat (v4.2.1) (62) R package was used for downstream analysis and visualization. Raw counts were log-normalized using the LogNormalize feature of Seurat, and the top 2,000 highly variable genes were selected as anchor features. Batch effects among samples were removed using reciprocal principal component analysis. Principal component analysis of the integrated data identified the top 10 principal components, which were subsequently used for dimensionality reduction and clustering, resulting in 4 cell clusters (C1–C4).

### Identification of signature genes and subsequent gene ontology analysis.

Cluster-specific signature genes were identified using the Wilcoxon rank-sum test with the following thresholds: log_2_(fold change) greater than 0.25, minimum percentage of expressed cells greater than 0.1, and an adjusted *P* value less than 0.05. Gene ontology analysis was performed using DAVID (63), as previously described.

### Sources of surface marker genes and secreted protein genes.

Lists of cell surface genes and secreted protein genes were obtained from The Human Protein Atlas (64) and screened based on “evidence in protein level.” The genes analyzed in this study are listed in Supplemental Tables 5 and 6.

### Pseudotime trajectory analysis.

Monocle (v2.22.0) (65) was used to determine the differentiation trajectory of erythroid cells across C1–C4. The log-normalized expression matrix and annotation profiles generated by Seurat were used as input, with a minimum expression level of 0.5 defined as true expression. Genes with high dispersion across cells were selected for ordering. Dimensionality reduction was performed using the discriminative dimensionality reduction with trees (DDRTree) method. The root of the trajectory was manually set based on hemoglobin gene expression.

### Cell-cell interaction analysis.

CellChat (v1.6.1) (66) was used to predict potential cell-cell communication from the log-normalized expression profiles generated by Seurat. The CellChatDB.mouse database was used to identify significant interactions, with only connections involving more than 10 cells in specific cell groups retained for further analysis. The significantly interacting cell pairs (*P* < 0.05) were selected and visualized.

### Bulk RNA-Seq and identification of DEGs.

After confirming AML cell purity by flow cytometry (>99%), purified AML cells from both monoculture and coculture systems were harvested for RNA-Seq. Total RNA was extracted from these cells using TRIzol reagent. The resulting RNA samples were sent to Novogene for library preparation and sequencing. Trimmomatic (v0.36) (67) was employed to filter out sequences with adapters and of low quality, obtaining clean reads. The filtered clean reads were aligned to the reference genome using STAR (v2.6.1d) (68). The read counts were then transformed into fragments per kilobase of transcript per million mapped reads (FPKM), and all subsequent analyses were performed using R software. DEGs were identified using DESeq2 (v1.34.0) as previously described (69).

### Gene set enrichment analysis.

Gene set enrichment analysis was performed using the software (v4.0.1) and gene sets obtained from MSigDB (70). The gene expression matrix was used as input data, with the number of permutations set to 1,000 and the permutation type set to “phenotype.” To determine statistically significant enrichment, a *P* value threshold of less than 0.05 was applied.

### WGCNA.

WGCNA was performed to identify the genes highly correlated among all meta-genes across different culture conditions. The normalized gene expression matrix from AML cells cultured alone or cocultured with CD81^−^ Erys or CD81^+^ Erys was used as input for WGCNA, implemented using the WGCNA R package (v1.73) (71). A soft-threshold power was selected using the scale-free topology criterion, and a signed adjacency matrix was constructed to identify coexpression modules. Gene module–trait relationships were assessed by correlating module eigengenes (ME) with different culture conditions. In WGCNA, each module is assigned a color label, and the ME represents the overall gene expression profile. “MEgrey” refers to genes that do not cocluster with others and are biologically uninformative. “MEgrey60” is a distinct functional module automatically labeled by WGCNA’s algorithm. Modules significantly associated with the CD81^+^ Erys coculture group (*P* < 0.05, module-trait relation score > 0.85) were further analyzed for functional enrichment using KEGG pathway analyses. KEGG pathway enrichment for each WGCNA module was performed using the DAVID database (63), with genes from each individual module as input. Significance was defined as an adjusted *P* value less than 0.05.

### Metabolomic profiling and analysis.

To investigate the metabolic alterations induced by CD81^+^ Erys coculture, AML cells (NT or *Egln3*_KO) were cultured under 3 different conditions: cocultured with CD81^+^ Erys, cocultured with CD81^−^ Erys, or cultured alone. After 9 days of culture, 1 × 10^5^ AML cells were purified and lysed to extract intracellular metabolites using 40 μL prechilled 80% methanol solution, which were then centrifuged; the supernatant was collected for liquid chromatography–mass spectrometry analysis.

Liquid chromatography was performed on an Xbridge BEH amide HILIC column (Waters) with a Vanquish UHPLC system (Thermo Fisher Scientific). Solvent A was 95:5 water/acetonitrile with 20 mM ammonium acetate and 20 mM ammonium hydroxide at pH 9.4. Solvent B was acetonitrile. The gradient used for metabolite separation was 0 minutes, 90% B; 2 minutes, 90% B; 3 minutes, 75% B; 7 minutes, 75% B; 8 minutes, 70% B; 9 minutes, 70% B; 10 minutes, 50% B; 12 minutes, 50% B; 13 minutes, 25% B; 14 minutes, 25% B; 16 minutes, 0% B; 21 minutes, 0% B; 22 minutes, 90% B; and 25 minutes, 90% B. Mass spectrometry analysis was performed on an Orbitrap Exploris 480 mass spectrometer (Thermo Fisher Scientific) by electrospray ionization with parameters as follows: scan mode, SIM m/z 50–900; spray voltage, 3.6 kV (positive) and −3.2 kV (negative); capillary temperature, 320°C; sheath gas, 40 arb; auxiliary gas, 7 arb; resolution, 120,000 (full mass spectrometry). The intensity of metabolites was then normalized by sum, log-transformed, and auto-scaled using the MetaboAnalyst platform (72). Missing values were replaced by limits of detection, defined as 1/5 of the minimum positive value for each variable. Differentially abundant metabolites were identified using Welch’s *t* test combined with FDR correction.

### Western blot.

Cells were lysed on ice using RIPA buffer (MilliporeSigma, R0278) with protease inhibitor cocktail (MilliporeSigma, P8340), mixed with 5× SDS-PAGE loading buffer and boiled for protein denaturation. The proteins were separated by SDS-PAGE, transferred to 0.45 μm PVDF membranes, blocked with 5% nonfat milk, and then probed with the following primary antibodies: PHD3 rabbit monoclonal antibody (1:1,000, Abcam, ab184714), LPCAT1 rabbit polyclonal antibody (1:2,000, Proteintech, 16112-1-AP), LPCAT3 rabbit monoclonal antibody (1:2,000, Proteintech, 67882-1-Ig), LPCAT4 rabbit polyclonal antibody (1:2,000, Proteintech, 17905-1-AP), and β-actin mouse monoclonal antibody (1:5,000, Proteintech, 66009-1-Ig). After incubation with the appropriate HRP-conjugated secondary antibodies, protein bands were visualized using enhanced chemiluminescence (Thermo Fisher Scientific, 34580) and imaged with a chemiluminescence detection system.

### Measurement of intracellular ROS levels.

We quantified intracellular ROS levels in AML cells under 3 conditions: (a) culture alone, (b) coculture with CD81^–^ Erys, and (c) coculture with CD81^+^ Erys using CellROX deep red reagent (Thermo Fisher Scientific, C10491). Briefly, at the designated time point, cells were harvested and incubated with the CellROX probe at a final concentration of 5 μM for 30 minutes at 37°C protected from light. After washing, the MFI of the CellROX signal was analyzed by flow cytometry specifically on the gated AML cell population.

### Statistics.

GraphPad Prism software (v9.4.0) was used for statistical analysis and data visualization. Quantitative values are expressed as mean ± SEM, with each data point representing independent observations. Significance levels were determined through various statistical tests, including 2-tailed unpaired *t* test for 2-group comparisons, 1-way ANOVA for multiple group comparisons, and a log-rank test for survival analysis. A *P* value of less than 0.05 was considered significant.

### Study approval.

All animal procedures were conducted in strict accordance with established animal care guidelines and approved by the IACUCs of the State Key Laboratory of Experimental Hematology and the Institute of Hematology and Blood Diseases Hospital.

### Data availability.

The transcriptomics data generated in this study are publicly available in the Genome Sequence Archive under accession numbers CRA016087 and CRA016079. All primary metabolomics data have been submitted to Figshare (https://doi.org/10.6084/m9.figshare.30265513.v1). Values for all data points in graphs are reported in the Supporting Data Values file.

## Author contributions

LS, H Cheng, TC, and LW supervised the study; YL, JC, and PT performed the functional experiments with the help of GS, ZY, X Zang, FD, SY, JG, X Zhao, JM, DW, and LZ; JT and YL performed the bioinformatics analyses with the help of LS, H Cheng, and TC; H Chen and LW conducted the metabolomic profiling; YL, JC, JT, and PT analyzed the data; YL, JC, JT, PT, LW, TC, H Cheng, and LS wrote the manuscript. All authors discussed the results and reviewed the manuscript. Authorship order among co–first authors is ranked based on the level of contribution to the project.

## Funding support

- National Key Research and Development Program of China (2022YFA1103503 to LS and 2024YFC2510500 to JG).
- National Natural Science Foundation of China (82225003 to LS, 82525002 to HC, 92468206 to HC, 82100152 to JT, U24A20749 to JT, 82300141 to JG, and 82400148 to JM).
- CAMS Innovation Fund for Medical Sciences (2024-I2M-TS-036 to LS, 2021-I2M-1-040 to TC, 2023-I2M-2-007 to HC, 2021-I2M-1-073 to JT, and 2023-I2M-2-007 to LS and HC).
- Haihe Laboratory of Cell Ecosystem Innovation Fund (HH24KYZX0005 to LS, 24HHXBSS00006 to HC, and HH23KYZX0004 to TC).
- Tianjin Municipal Science and Technology Commission Grant (24ZXRKSY00010 to LS, 24ZXZSSS00080 to JT, and 23ZXRKSY00010 to LS).
- Nonprofit Central Research Institute Fund of Chinese Academy of Medical Sciences (2021-RC350-008 to LW).

## Supplementary Material

Supplemental data

Unedited blot and gel images

Supplemental table 1

Supplemental table 2

Supplemental table 3

Supplemental table 4

Supplemental table 5

Supplemental table 6

Supplemental table 7

Supplemental table 8

Supplemental table 9

Supplemental table 10

Supplemental table 11

Supplemental table 12

Supplemental table 13

Supplemental table 14

Supporting data values

## Version 1 12/15/2025

Electronic publication
