# Supplementary material for: Leukemia-expanded splenic CD81+ erythroblasts potentiate disease progression in mice by reshaping leukemic cell metabolism
Source: J Clin Invest. 2025 Dec 15;135(24):e193082. doi: 10.1172/JCI193082 (PMC12700549; doi:10.1172/JCI193082)
Supplement: Supplemental data [file jci-135-193082-s202.pdf]

Supplemental Figures

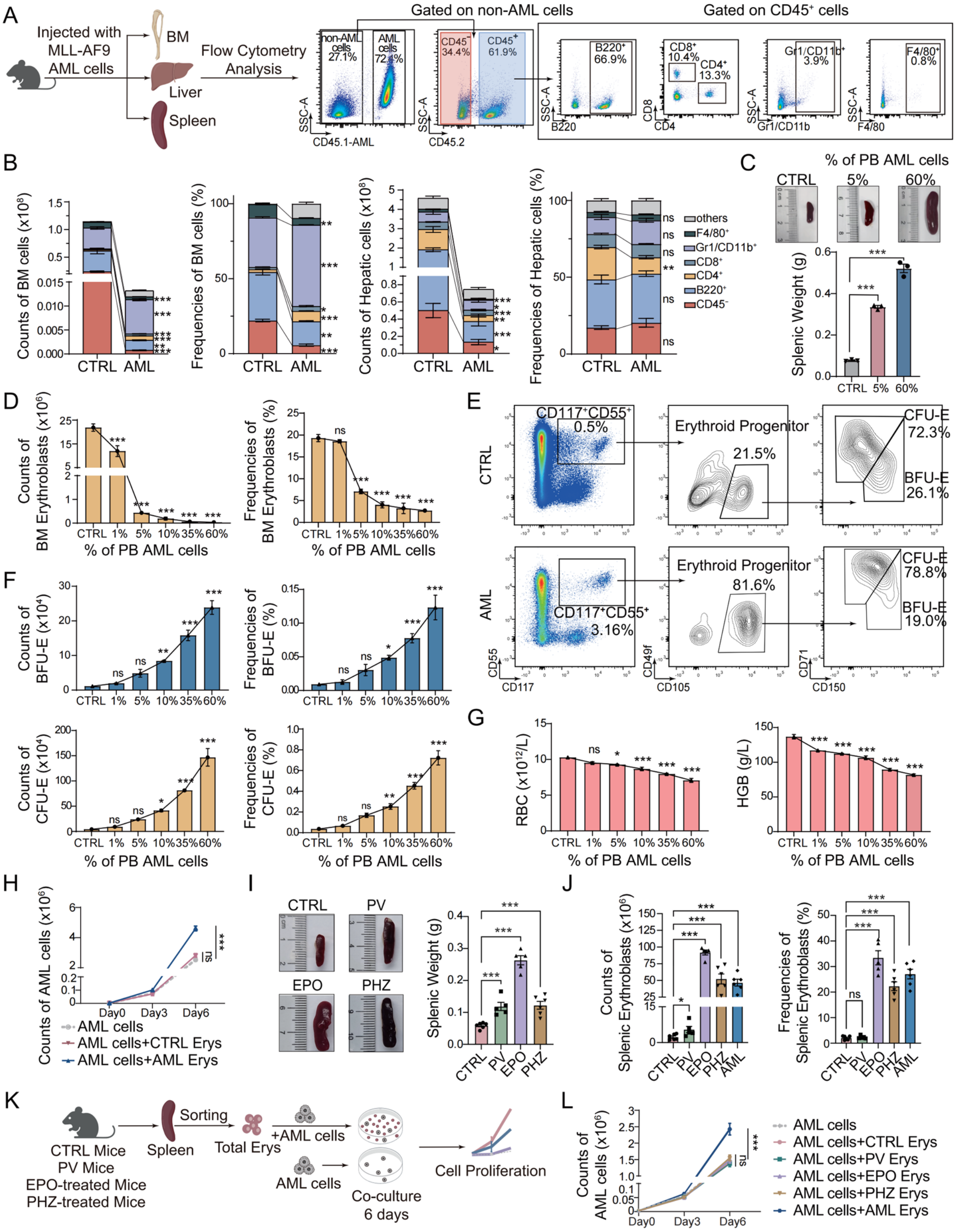

Supplemental Figure 1. An erythroblast population is markedly expanded in the spleens of AML mice. (A) A schematic illustrating the experimental design for analyzing BM, liver, and spleen samples from control (CTRL) mice and mice with advanced AML by flow cytometry, gating on CD45⁻ cells, B220⁺ B cells, CD4⁺ T cells, CD8⁺ T cells, Gr1/CD11b⁺ myeloid cells, F4/80⁺ macrophages, and other non-AML cell populations. (B) Cell counts and frequencies of CD45⁻ cells, B220⁺ B cells, CD4⁺ T cells, CD8⁺ T cells, Gr1/CD11b⁺ myeloid cells, F4/80⁺ macrophages, and other non-AML cell populations in the BM and liver of CTRL mice and mice with advanced AML (n=3). (C) Photographs (top) and weights (bottom) of the spleens from CTRL mice and AML mice (with 5% and 60% AML cell infiltration in the PB) (n=3). (D) Cell counts and frequencies of CD45⁻CD71⁺Ter119⁺CD44⁺ erythroblasts in the BM of CTRL mice

and AML mice at different disease stages (n=3). (E) Representative flow cytometry showing the gating strategies used to analyze BFU-E and CFU-E from spleens of CTRL mice and AML mice. (F) Cell counts and frequencies of BFU-E and CFU-E in the spleens of CTRL mice and AML mice at different disease stages (n=3). (G) Red blood cell (RBC) counts and hemoglobin (HGB) concentrations in CTRL mice and AML mice at different disease stages (n=3). (H) Proliferation curves of AML cells cultured alone or co-cultured with $CD45^{-}CD71^{+}Ter119^{+}CD44^{+}$ erythroblasts isolated from the spleens of CTRL mice or mice with advanced AML (n=3). (I) Photographs (left) and weights (right) of the spleens from CTRL, polycythemia vera (PV), erythropoietin (EPO)-treated, and PHZ-treated mice (n≥5). For the PV model, *JAK2*^{V617F} transgenic mice (PB HGB>190 g/L) were used. For PHZ treatment, mice received intraperitoneal injections of PHZ (100 mg/kg) and were sacrificed on Day 4. For EPO treatment, mice received intraperitoneal injections of EPO (50 U/day for 5 days) and were sacrificed on Day 6. (J) Cell counts and frequencies of $CD45^{-}CD71^{+}Ter119^{+}CD44^{+}$ erythroblasts in the spleens of CTRL, PV, EPO-treated, PHZ-treated, and AML mice (n≥5). (K) A schematic illustrating the experimental design for co-culturing AML cells with $CD45^{-}CD71^{+}Ter119^{+}CD44^{+}$ erythroblasts sorted from the spleens of CTRL, PV, EPO-treated, and PHZ-treated mice for 6 days. AML cells cultured alone served as controls. (L) Proliferation curves of AML cells cultured alone or co-cultured (n=4). Data are presented as the mean ± SEM. The results shown are representative of one of three independent experiments with consistent trends. Statistical analyses were performed using a two-tailed unpaired t-test (B, I, J) or one-way ANOVA (C, D, F, G, H, L). *P<0.05, **P<0.01, ***P<0.001; ns, not significant.

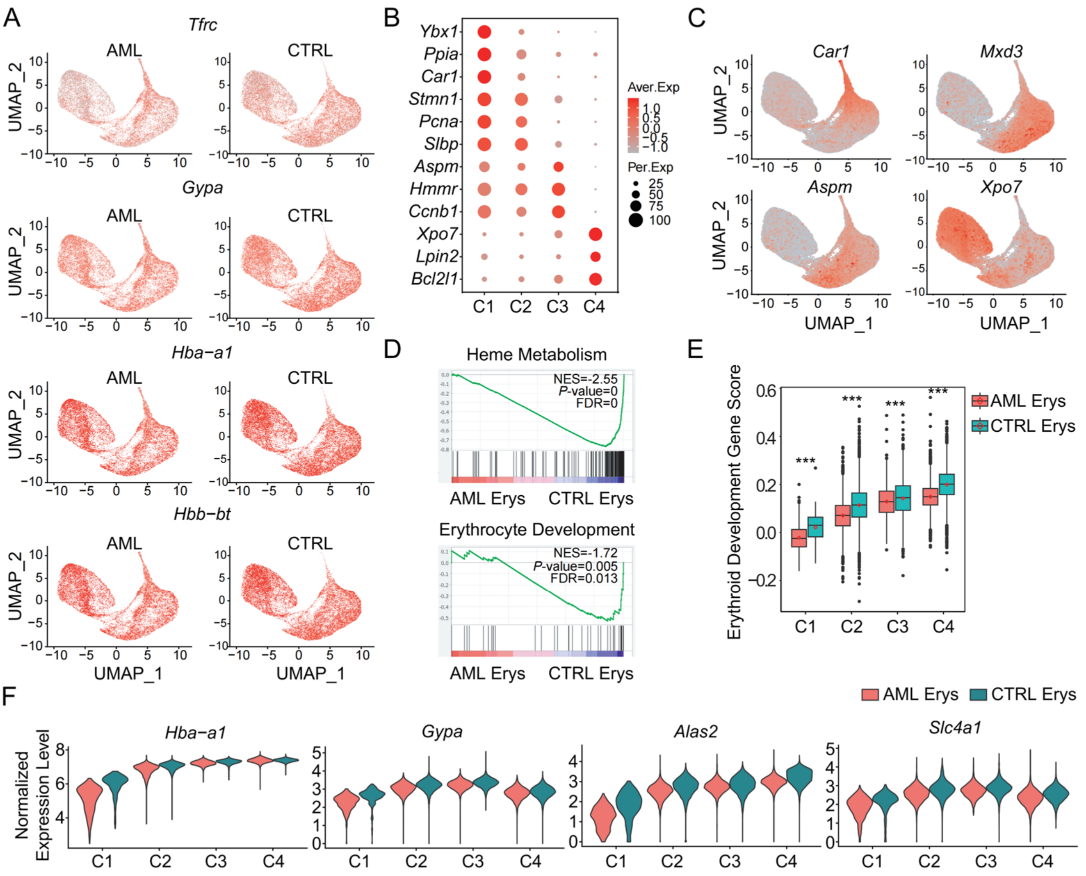

Supplemental Figure 2. AML Erys and CTRL Erys possess different characteristics. (A) UMAP visualization showing the expression levels of four erythroid-lineage-specific genes (*Tfrc*, *Gypa*, *Hba-a1*, *Hbb-bt*) across different conditions. (B) Representative signature genes of C1–C4 clusters. (C) UMAP visualization showing the expression levels of four stage-specific representative genes (*Car1*, *Mxd3*, *Aspm*, *Xpo7*) of terminal erythroid differentiation. (D) GSEA plot showing enriched pathways between AML Erys and CTRL Erys, based on ranked gene expression using the HALLMARK heme metabolism and GO erythrocyte development (GO:0048821). Positive enrichment scores indicate upregulation in AML Erys. NES (normalized enrichment score), *P*-value and FDR are shown. (E) Box plot showing expression scores of genes associated with erythrocyte development (GO:0048821) in AML Erys and CTRL Erys across C1–C4. (F) Violin plot showing expression of key genes related to erythrocyte differentiation (*Hba-a1*, *Gypa*, *Alas2*, *Slc4a1*). Statistical analyses were performed using the two-tailed Wilcoxon rank-sum test (E). ***P<0.001.

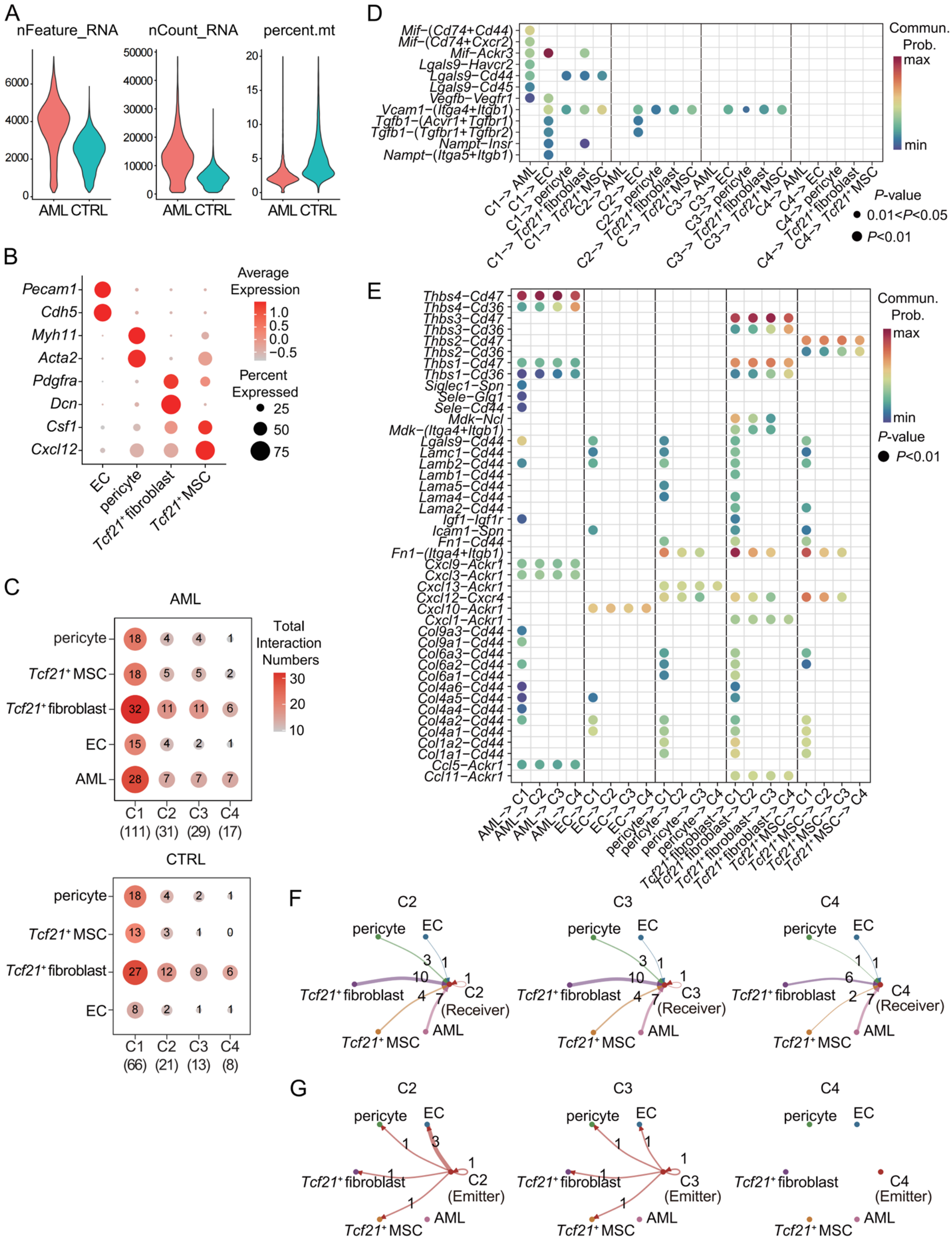

Supplemental Figure 3. AML C1 Erys exhibit stronger interactions with surrounding cells in the splenic AML microenvironment. (A) The quality metrics across $CD45^{-}Ter119^{-}CD71^{-}$ splenic stromal cells from AML or CTRL, with uniform distributions of nFeature_RNA (detected genes per cell), nCount_RNA (UMIs per cell) and percent.mt (mitochondrial gene content). (B) Dot plot showing representative signature genes of endothelial cells (ECs), pericytes, *Tcf21*$^{+}$ fibroblasts, and *Tcf21*$^{+}$ mesenchymal stem cells (MSCs) in the splenic AML microenvironment. Dot size represents the percentage of cells expressing the gene; color indicates average expression. (C) Total interaction numbers between C1–C4 Erys (from AML or CTRL) and other cell populations within the splenic microenvironment. (D) Ligand-receptor pairs mediating signals received by other cell populations from AML C1–C4 Erys in the splenic AML microenvironment, with communication probability and significance (*P*-value). (E) Ligand-receptor pairs mediating signals emitted by other cell populations to AML C1–C4 Erys in the splenic AML microenvironment. (F and G) Directional network plots showing the number of incoming (F) and outgoing (G) interactions for AML C2–C4 Erys and other cells in the AML microenvironment.

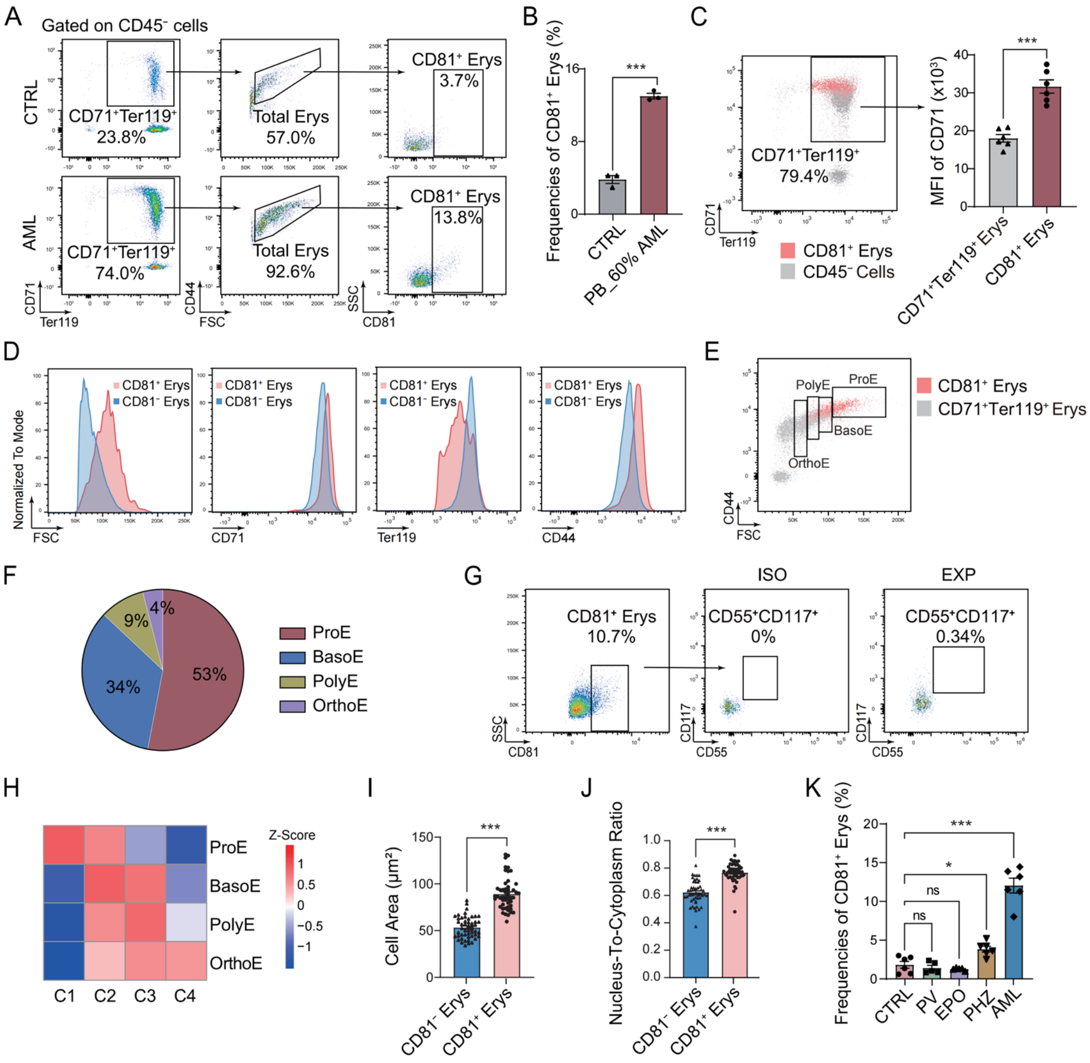

Supplemental Figure 4. Characteristics of CD81$^+$ Erys. (A) Gating strategies used to isolate CD81$^+$ Erys from the spleens of CTRL and AML mice. (B) Percentage of CD81$^+$ Erys within the population of Total Erys (CD71$^+$TER119$^+$CD44$^+$) in CTRL mice or mice with advanced AML (n=3). (C) Representative flow cytometry plot showing the expression of CD71 on CD81$^+$ Erys (left) and the median fluorescence intensity (MFI) of CD71 on CD71$^+$TER119$^+$ Erys and CD81$^+$ Erys (right) (n=6). (D) Flow cytometry histograms comparing size (forward scatter, FSC) and the expression of CD71, Ter119, and CD44 between CD81$^+$ Erys and CD81$^-$ Erys. (E) Representative flow cytometry plot showing the distribution of CD81$^+$ Erys within CD71$^+$Ter119$^+$ Erys. (F) Percentages of ProE, BasoE, PolyE and OrthoE subsets within the CD81$^+$ Erys population. (G) Representative flow cytometry dot plots showing the CD117$^+$CD55$^+$ population in CD81$^+$ Erys. (H) Correlation heatmap comparing C1–C4 with FACS-purified erythroid precursors at defined stages from the GSE53983 dataset. Pearson correlation coefficients were calculated using the average gene expression of each cluster and 1,000 highly variable genes associated with erythroid differentiation. Values were row-scaled to highlight relative similarities within each erythroid stage. Warmer colors indicate higher relative correlation. (I and J) Cell areas (I) and nucleus-to-cytoplasm ratios (J) of CD81$^-$ Erys and CD81$^+$ Erys isolated from the spleens of mice with advanced AML (n=50). (K) Frequencies of CD81$^+$ Erys among CD45$^-$CD71$^+$Ter119$^+$CD44$^+$ Erys in the spleens of CTRL, PV, EPO-treated, PHZ-treated, and AML mice (n≥5). Data are presented as the mean ± SEM. The results shown are representative of one of three independent experiments with consistent trends. Statistical analyses were performed using a two-tailed unpaired t-test (B, C, I, J), or one-way ANOVA (K). $^*P<0.05$, $^{***}P<0.001$; ns, not significant.

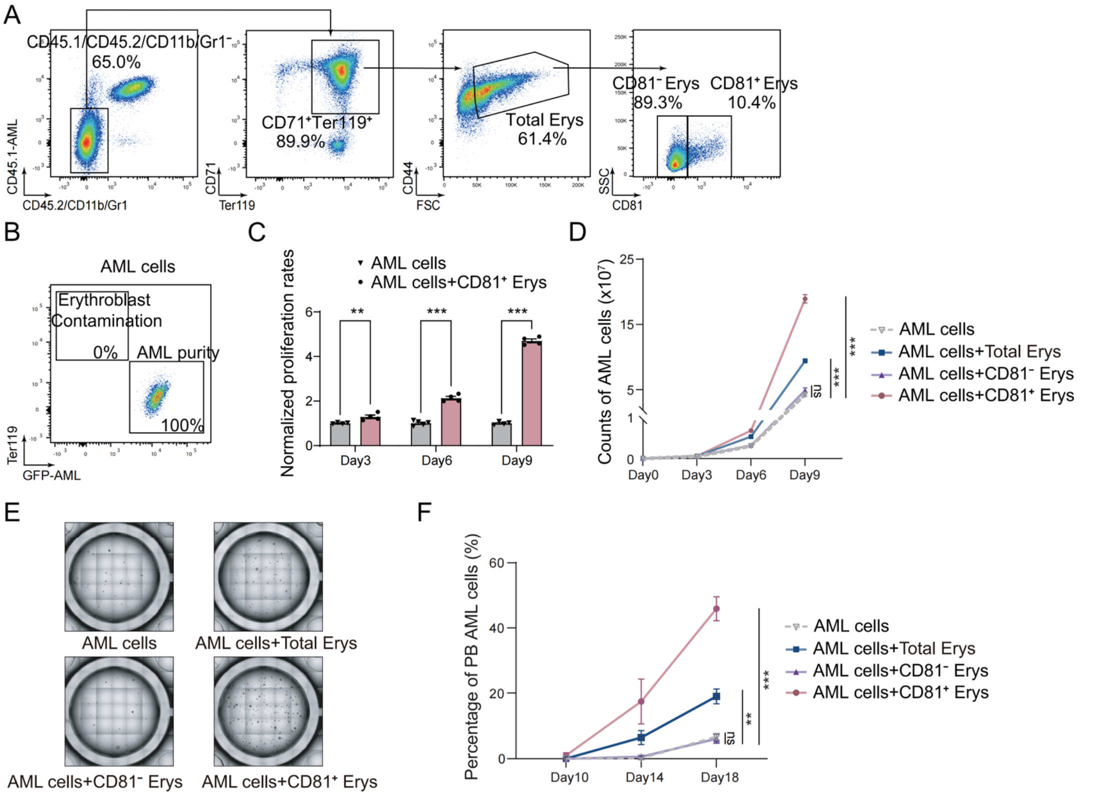

Supplemental Figure 5. CD81$^+$ Erys promote AML cell proliferation and contribute to disease progression. (A) Gating strategies used to isolate CD81$^-$ Erys and CD81$^+$ Erys from the spleens of AML mice. (B) Representative flow cytometry plots assessing the purity of AML cells at the start of the co-culture experiment. (C) Pro-proliferative effects of CD81$^+$ Erys on AML cells on Day 3, 6 and 9 (n=4). (D) Proliferation curves of AML cells cultured alone or co-cultured with Total Erys, CD81$^-$ Erys, or CD81$^+$ Erys (n=6). (E) Representative images of colonies formed by cultured AML cells. (F) AML progression rates in mice intravenously injected with cultured AML cells (n=8). Data are presented as the mean ± SEM. The results shown are representative of one of three independent experiments with consistent trends. Statistical analyses were performed using a multiple unpaired t-test (C), one-way ANOVA (D, F). ***P*<0.01, ****P*<0.001; ns, not significant.

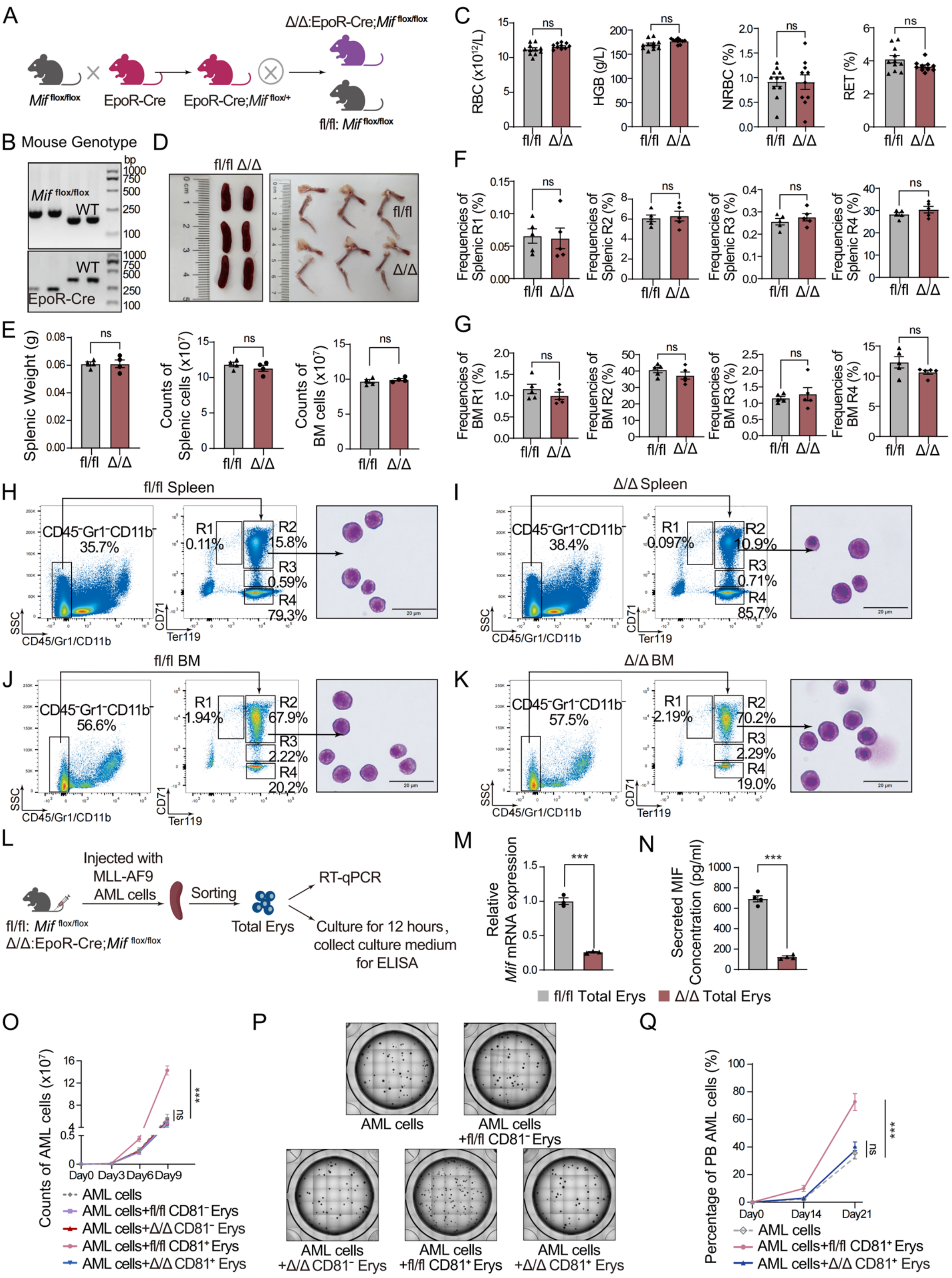

Supplemental Figure 6. MIF plays a central role in mediating the AML-promoting effects of CD81^{+} Erys. (A) A schematic illustrating the generation of EpoR-Cre;*Mif*$^{flox/flox}$ (Δ/Δ) mice by crossing *Mif*$^{flox/flox}$ mice with EpoR-Cre mice. (B and C) Mouse genotype (B) and various erythroid-related indicators in the PB (C) of *Mif*$^{flox/flox}$ (fl/fl) and Δ/Δ mice (n≥10). (D and E) Photographs of spleen and bone samples (n=3) (D), splenic weights, splenic cell counts, and BM cell counts (n=4) (E) of fl/fl and Δ/Δ mice. (F and G) Frequencies of splenic (F) and BM (G) R1–R4 populations in fl/fl and Δ/Δ mice (n=5). (H–K) Gating strategies for analyzing the proportions of CD71^{+}Ter119$^{low/-}$ (R1), CD71^{+}Ter119^{+} (R2), CD71$^{low/-}$Ter119^{+} (R3), and CD71^{-}Ter119^{+} (R4) erythroid cells in the fl/fl spleen (H), Δ/Δ spleen (I), fl/fl BM (J) and Δ/Δ BM (K), along with representative Wright-Giemsa staining images of sorted R2 erythroid cells. Scale bar, 20 μm. (L–N) A schematic illustrating the experimental design (L) for AML mouse model induction, flow cytometric sorting of Total Erys from the spleens of fl/fl and Δ/Δ mice, and confirmation of *Mif* depletion by RT-qPCR (n=3) (M) and ELISA (n=4) (N). (O) Proliferation curves of AML cells cultured alone or co-cultured with fl/fl CD81^{-} Erys, Δ/Δ CD81^{-} Erys, fl/fl CD81^{+} Erys, or Δ/Δ CD81^{+} Erys (n=6). (P)

Representative images of colonies formed by cultured AML cells. (Q) AML progression rates in mice intravenously injected with cultured AML cells (n=8). Data are presented as the mean ± SEM. The results shown are representative of one of three independent experiments with consistent trends. Statistical analyses were performed using a two-tailed unpaired t-test (C, E, F, G, M, N) or one-way ANOVA (O, Q). ***$P<0.001$; ns, not significant.

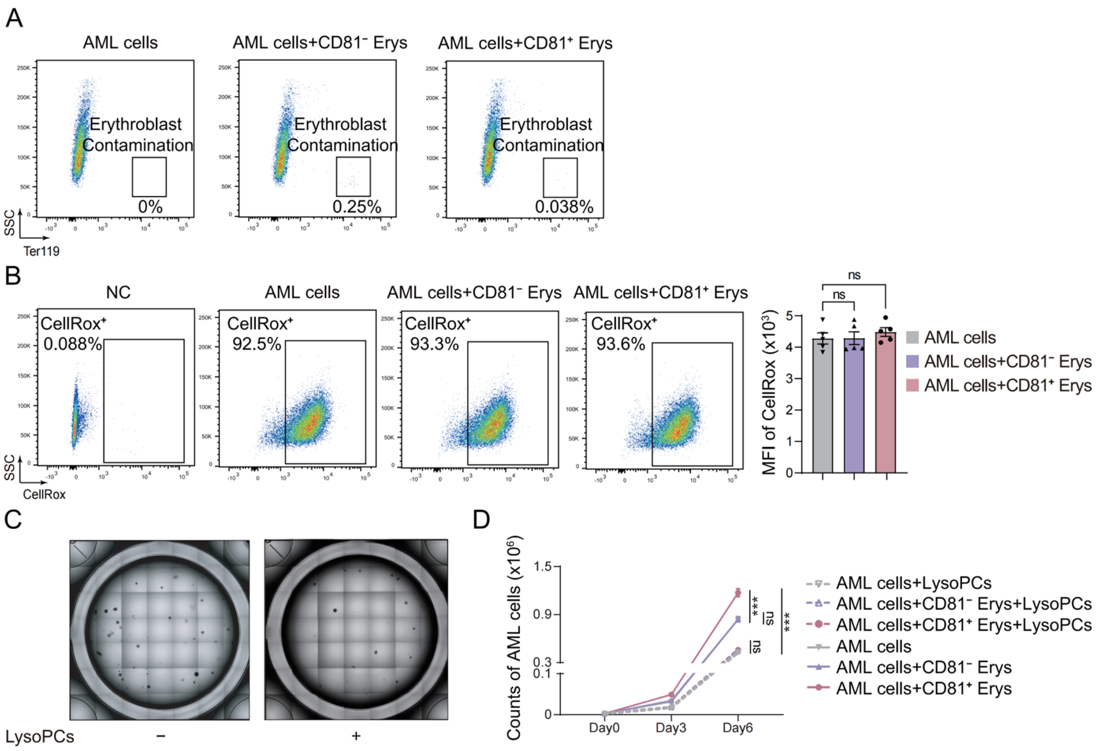

Supplemental Figure 7. Co-culturing with CD81⁺ Erys reshapes the metabolic profile of AML cells. (A) Representative flow cytometry plots assessing the purity of AML cells after 9 days of co-culture with erythroblasts. (B) Flow cytometry for quantifying reactive oxygen species (ROS) in AML cells cultured under three conditions: AML cells monoculture, AML cells+CD81⁻ Erys, and AML cells+CD81⁺ Erys. MFI of CellROX staining (n=5). (C) Representative images of colonies formed by AML cells treated with 10 μM lysophosphatidylcholines (LysoPCs) compared to controls. (D) Proliferation curves of AML cells cultured alone or co-cultured with CD81⁻ Erys or CD81⁺ Erys in the absence or presence of 10 μM LysoPCs (n=5). Data are presented as the mean ± SEM. The results shown are representative of one of three independent experiments with consistent trends. Statistical analyses were performed using one-way ANOVA (B, D). ****P*<0.001; ns, not significant.

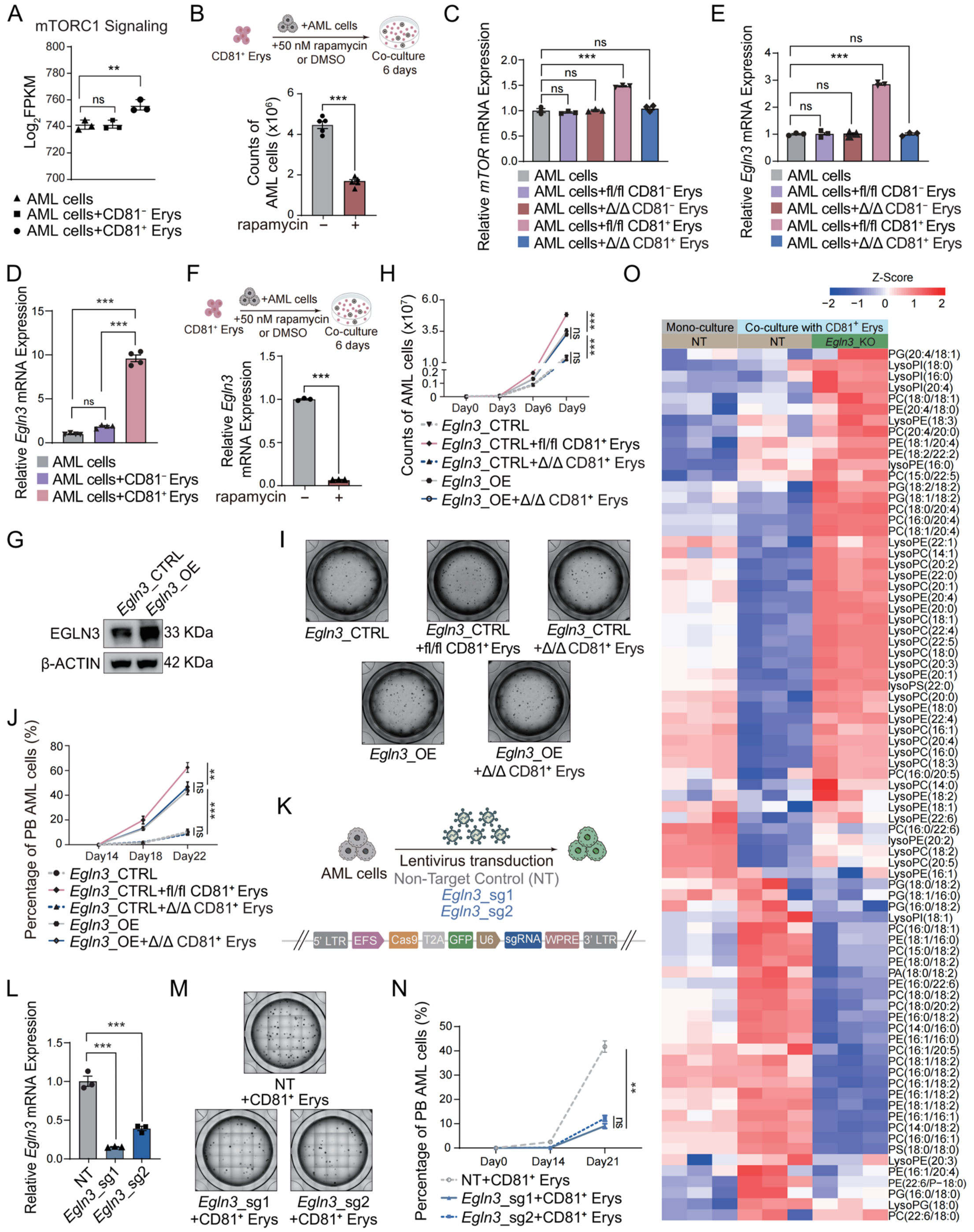

Supplemental Figure 8. MIF/CD74-mTORC1-EGLN3 regulatory axis rebalances lipid metabolism in AML cells during co-culture. (A) Expression of the mTORC1 signaling gene set in AML cells cultured alone or co-cultured with CD81⁺ Erys or CD81⁻ Erys (n=3). (B) A schematic illustrating the experimental design for co-culturing AML cells with CD81⁺ Erys in the presence of 50 nM rapamycin or DMSO control (top). Cell counts of AML cells obtained under each co-culture condition (n=5) (bottom). (C) Relative *mTOR* mRNA expression levels in AML cells after 9 days of culture alone or co-culture with fl/fl CD81⁻ Erys, Δ/Δ CD81⁻ Erys, fl/fl CD81⁺ Erys, or Δ/Δ CD81⁺ Erys (n=3). (D) Relative *Egln3* mRNA expression levels in AML cells after 9 days of culture alone or co-culture with CD81⁻ Erys or CD81⁺ Erys (n=4). (E) Relative *Egln3* mRNA expression levels in AML cells after 9 days of culture alone or co-culture with fl/fl CD81⁻ Erys, Δ/Δ CD81⁻ Erys, fl/fl CD81⁺ Erys, or Δ/Δ CD81⁺ Erys (n=3). (F) A schematic illustrating the experimental design for co-culturing AML cells with CD81⁺ Erys, in the presence of 50 nM rapamycin or DMSO (top). Expression of *Egln3* in AML cells obtained under each co-culture condition (n=3) (bottom). (G) Protein expression levels of EGLN3 in *Egln3*_CTRL or *Egln3*_OE AML cells. β-ACTIN was used as a loading control. Molecular weights are indicated on the right. (H) Proliferation curves of *Egln3*_CTRL or *Egln3*_OE AML cells cultured alone or co-cultured with Δ/Δ CD81⁺ Erys (n≥3). *Egln3*_CTRL AML cells

co-cultured with fl/fl $CD81^+$ Erys served as a positive control. (I) Representative images of colonies formed by cultured AML cells. (J) AML progression rates in mice intravenously injected with cultured AML cells (n=8). (K) A schematic illustrating the construction of NT and *Egln3*_KO (*Egln3*_sg1, *Egln3*_sg2) AML cells. (L) Relative *Egln3* mRNA expression levels in NT and *Egln3*_KO AML cells (n=3). (M) Representative images of colonies formed by NT and *Egln3*_KO AML cells co-cultured with $CD81^+$ Erys. (N) AML progression rates in mice intravenously injected with NT and *Egln3*_KO AML cells co-cultured with $CD81^+$ Erys (n=8). (O) Heatmap showing the differential metabolite profiles of LysoPLs and PLs in NT AML cells cultured alone, or NT and *Egln3*_KO AML cells co-cultured with $CD81^+$ Erys. Data are presented as the mean ± SEM. The results shown are representative of one of three independent experiments with consistent trends. Statistical analyses were performed using one-way ANOVA (A, C, D, E, H, J, L, N) or a two-tailed unpaired t-test (B, F). $^{**}P<0.01$, $^{***}P<0.001$; ns, not significant.
