# Supplementary material for: Leukemia-expanded splenic CD81+ erythroblasts potentiate disease progression in mice by reshaping leukemic cell metabolism
Source: J Clin Invest. 2025 Dec 15;135(24):e193082. doi: 10.1172/JCI193082 (PMC12700549; doi:10.1172/JCI193082)

Full unedited blot for Figure 6D

Lane 1: AML cells
Lane 2: AML cells+CD81⁻ Erys
Lane 3: AML cells+CD81⁺ Erys (as shown in Figure 6D).

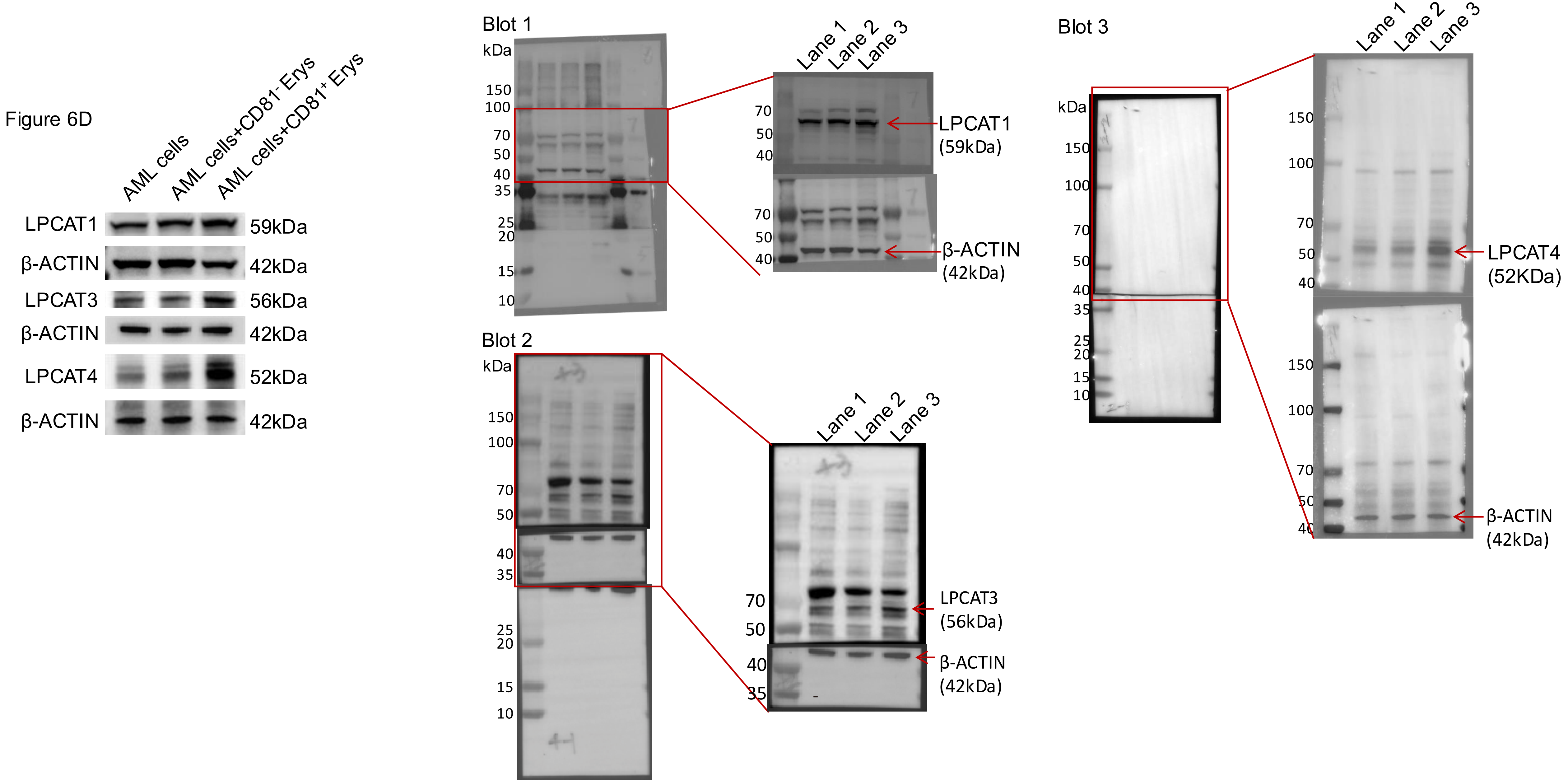

Full unedited blot for Supplemental Figure 8G

Lane 1: *Egln3*_CTRL
Lane 2: *EGLN3*_OE (as shown in Supplemental Figure 8G).

Supplemental Figure 8G

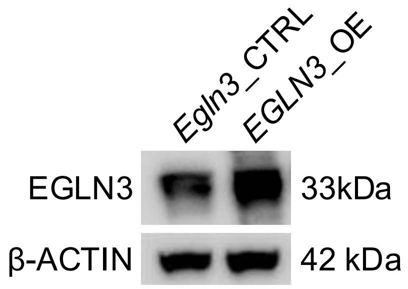

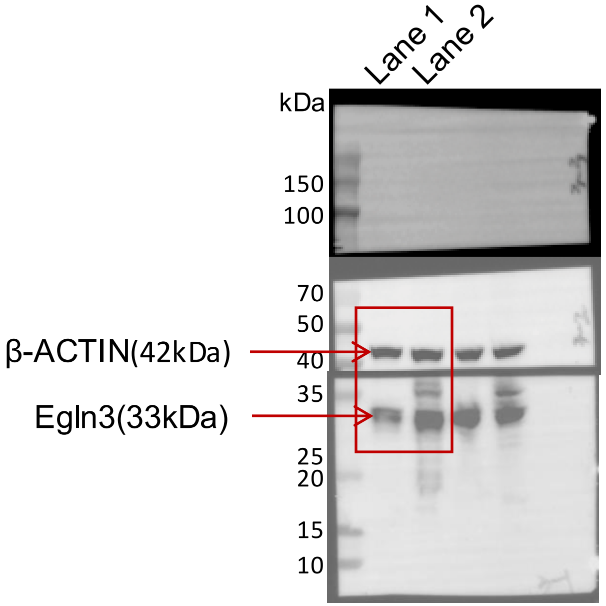

Full unedited gel for Supplemental Figure 6B

Supplemental Figure 6B (up)

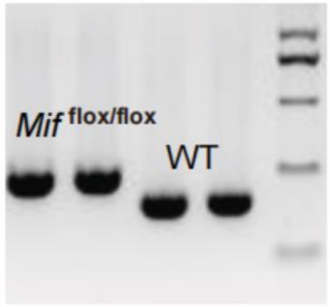

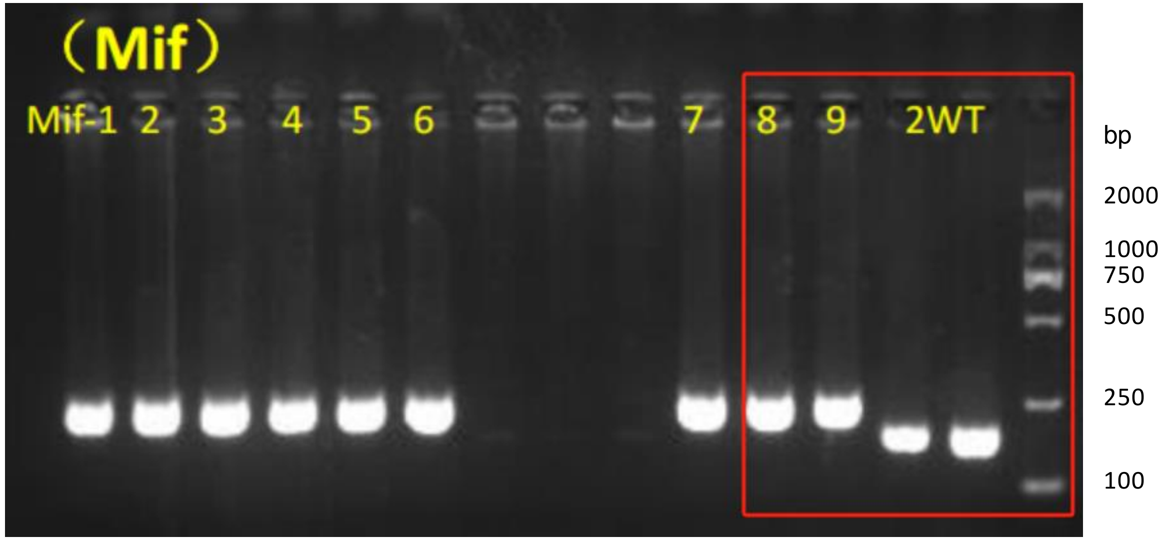

Supplemental Figure 6B (bottom)

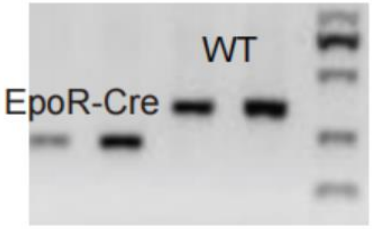

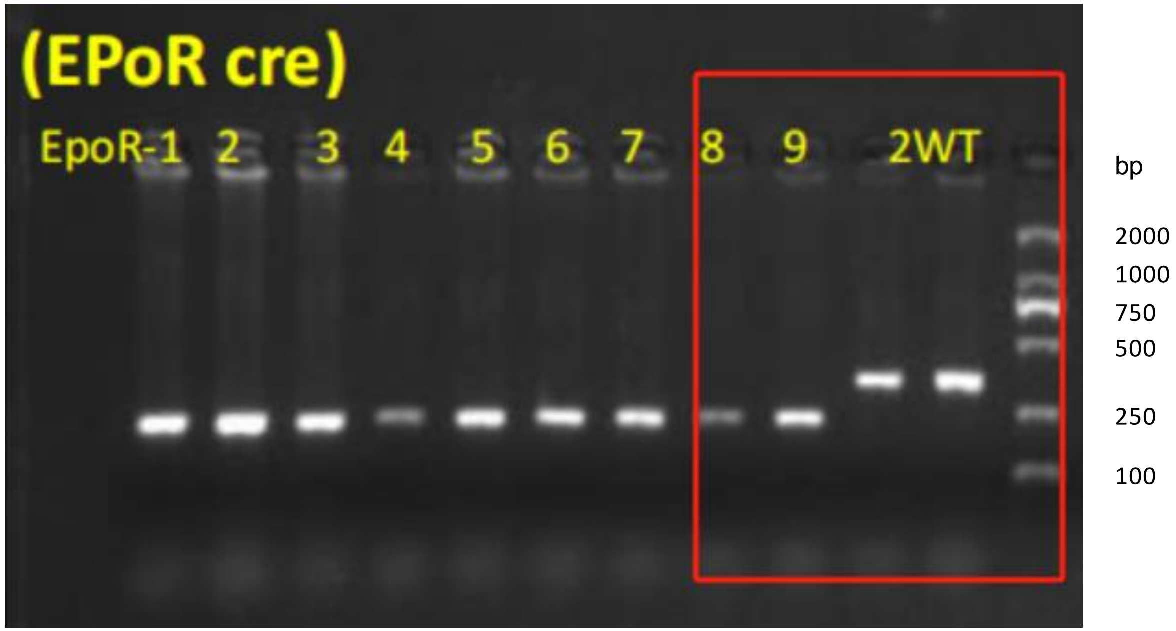

Supplement: Unedited blot and gel images [file jci-135-193082-s203.pdf]
